# *In vitro* effect of *Withania somnifera*, AYUSH-64, and remdesivir on the activity of CYP-450 enzymes: Implications for possible herb−drug interactions in the management of COVID-19

**DOI:** 10.3389/fphar.2022.973768

**Published:** 2022-10-12

**Authors:** Siva Swapna Kasarla, Swapnil P. Borse, Yashwant Kumar, Neha Sharma, Madhu Dikshit

**Affiliations:** ^1^ Translational Health Science and Technology Institute (THSTI), Faridabad, India; ^2^ Spatial Metabolomics Group, Leibniz-Institut für Analytische Wissenschaften-ISAS-e V, Dortmund, Germany; ^3^ AYUSH - Center of Excellence (AYUSH-CoE), Center for Complementary and Integrative Health [CCIH], Interdisciplinary School of Health Sciences (ISHS), Savitribai Phule Pune University Pune (SPPU), Pune, India; ^4^ Department of Pharmaceutical Analysis, Delhi Pharmaceutical Science and Research University, Pushp Vihar, New Delhi, India; ^5^ CSIR- Central Drug Research Institute, Lucknow, Uttar Pradesh, India

**Keywords:** *Withania somnifera*, Ayurveda, remdesivir, integrative medicine, Rasayana, LC-MS/MS, AYUSH-64, herb−drug interaction

## Abstract

Ayurvedic medicines *Withania somnifera* Dunal (*ashwagandha*) and AYUSH-64 have been used for the prevention and management of COVID-19 in India. The present study explores the effect of *Ashwagandha* and AYUSH-64 on important human CYP enzymes (CYP3A4, CYP2C8, and CYP2D6) to assess their interaction with remdesivir, a drug used for COVID-19 management during the second wave. The study also implies possible herb−drug interactions as *ashwagandha* and AYUSH-64 are being used for managing various pathological conditions. Aqueous extracts of *ashwagandha* and AYUSH-64 were characterized using LC-MS/MS. A total of 11 and 24 phytoconstituents were identified putatively from *ashwagandha* and AYUSH-64 extracts, respectively. In addition, *in silico* studies revealed good ADME properties of most of the phytoconstituents of these herbal drugs and suggested that some of these might possess CYP-450 inhibitory activity. *In vitro* CYP-450 studies with human liver microsomes showed moderate inhibition of CYP3A4, 2C8, and 2D6 by remdesivir, while *ashwagandha* had no inhibitory effect alone or in combination with remdesivir. AYUSH-64 also exhibited a similar trend; however, a moderate inhibitory effect on CYP2C8 was noticed. Thus, *ashwagandha* seems to be safe to co-administer with the substrates of CYP3A4, CYP2C8, and CYP2D6. However, caution is warranted in prescribing AYUSH-64 along with CYP2C8 substrate drugs. Furthermore, preclinical and clinical PK studies would be helpful for their effective and safer use in the management of various ailments along with other drugs.

## Graphical Abstract

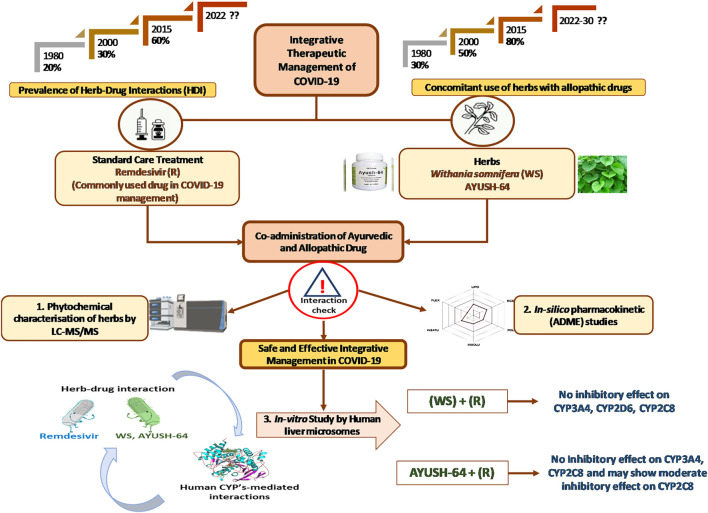

## Introduction

The modern system of medicine has emerged as the primary choice for the treatment of most of the health-related issues (Rakel, 2018). On the other hand, traditional, complementary, and alternative medicine (TCAM or CAM), like Ayurveda, Siddha, Unani, Sowa-Rigpa, and Chinese medicine, has gained popularity due to the history of their prolonged human use, efficacy, and higher safety (Borse et al., 2019). Currently, available treatment modalities of modern medicine and CAM are still contending to treat non-communicable and multi-factorial diseases like cancer, diabetes, and arthritis and also for the management of COVID-19 (Rakel, 2018; Borse et al., 2019). Therefore, the optimal and integrative use of both medical systems is required for the better management of human health. (Ye et al., 2021a; Zhang et al., 2021; Zhang et al., 2022). Patients of chronic illnesses intentionally or unintentionally use combinational/multimodal therapy; however, in the absence of data on potential herb−drug interactions (HDIs), their safety might be compromised (Fugh-Berman, 2000). The HDI studies on Chinese systems of medicine formulations have been recently conducted for the management of COVID-19; similarly, studies on Ayurvedic formulations that are being used worldwide in one and the other form must be explored (Ye et al., 2021a; Zhang et al., 2021; Zhang et al., 2022).

The World Health Organization (WHO) data and existing literature show that more than 70–80% world population use CAM for their healthcare needs. Particularly, in Western countries, CAM has become increasingly popular over the last few decades (Rakel, 2018; Borse et al., 2019). The health-seeking behavior studies from various parts of the world suggest widespread use of TCAM for the management of non-communicable diseases (Gordon et al., 2019; Hung et al., 2020; Toukabri et al., 2020). The pattern is almost similar in both developed and developing countries. Many provinces do not have evidence-based policies or regulations to rationalize the concomitant use of multiple modes of therapies (Fugh-Berman, 2000). Thus, systematic scientific investigations are required, especially to predict herb−drug interactions. Our study is particularly important in the Indian scenario as patients consume both herbal drugs and modern medicines. However, concomitant usage of herbs and conventional medicines could be much higher as healthcare professionals in Western countries might not ask the patients about the consumption of herbal remedies during prescriptions. Often patients also do not share the information pertaining to the consumption of herbal drugs (Fugh-Berman, 2000; Borse et al., 2019). Indeed, concomitant usage of herbs/CAM and conventional medicines have the potential issue of HDIs, which emerges as a major concern for the integrative medicine (IM) (Rees and Weil, 2001).

IM refers to the blending of conventional and evidence-based complementary medicines with the aim of using the most appropriate modalities for efficient patient care (Rees and Weil, 2001; Wainapel et al., 2015; Borse et al., 2019). *Ashwagandha* is a well-known *Rasayana* (∼rejuvenator) also referred to as “Indian ginseng” and is well known for its immunomodulatory, adaptogenic, anti-cancer, anti-diabetic, and anti-COVID-19 activities (Gogte, 2000; Winters, 2006). AYUSH-64 is a polyherbal formulation containing the aqueous extracts of *Rasayana* botanicals, namely, Saptaparna bark (*Alstonia scholaris* R. Br.), Katuki roots (*Picrorhiza kurroa* Royle ex. Benth), *Kiratatikta* whole-plant (*Swertia Chirata* Pexbex. Karst), and Kuberaksha seed (*Caesalpinia crista* L.). AYUSH-64 formulation has been known for its anti-malarial activity since 1994 (Gundeti et al., 2020b). Recently, it has been extensively explored for the integrative management of COVID-19 (Bhapkar et al., 2020; Chaturvedi et al., 2020; Patwardhan et al., 2020; Borse et al., 2021; Chopra et al., 2021; Kotecha, 2021; Saggam et al., 2021). In addition, these herbal formulations are also being used as home remedies to combat various communicable and non-communicable diseases (Bhapkar et al., 2020; Chaturvedi et al., 2020; Patwardhan et al., 2020; Borse et al., 2021; Chopra et al., 2021; Kotecha, 2021; Saggam et al., 2021). Patients with chronic illnesses use combination therapy, with or without consulting their physicians, which might result in serious HDIs. It is already known that HDIs might be beneficial, harmful, or even fatal (Babos et al., 2021) and are also noticed during various pharmacodynamics and pharmacokinetic (at any stage of ADME) studies (Fasinu et al., 2012). Most of the xenobiotics and herbal drugs are known to be metabolized by phase I enzymes rather than phase II ones. Cytochrome P450-mediated phase I metabolizing enzymes account for the xenobiotic transformation of 90% of drugs and herbal medicines (Ogu and Maxa, 2000; Rosenkranz et al., 2012). In order to anticipate the possible drug interactions for better therapeutic and safety profiles of IM, it is necessary to investigate the metabolic interactions of both allopathic and herbal drug candidates (Patwardhan, 2010). Despite 60 CYP isoforms that are predicted on the basis of the human genome, six CYP enzymes, namely, 1A2, 2C8, 2C9, 2C19, 2D6, and 3A4 are majorly involved in the metabolism of 70–90% of drugs (Rosenkranz et al., 2012).

The present study, therefore, investigates the probable HDIs associated with the usage of *ashwagandha* and AYUSH-64 in the management of COVID-19. In this study, we performed the phytochemical characterization of *ashwagandha* and AYUSH-64 by LC-MS/MS. *In silico* pharmacokinetic (ADME) parameters were assessed with the known phytoconstituents from both the herbal formulations. In addition, we also used remdesivir (an anti-viral drug used for COVID-19 management) as a representative example for HDI studies. Remdesivir, a ProTide (a prodrug of nucleotide), is diffused into the cells and is converted to a stable metabolite GS-441524 *via* an intermediate GS704277 (Abu Samaan et al., 2019). As per the fact sheet from the US FDA for healthcare providers and the summary for compassionate use by the European Medicines Agency (EMA), remdesivir is a substrate for CYP3A4, 2C8, and 2D6. Remdesivir also inhibits CYP3A4, while it has no effect on CYP1A1, 1A2, 2B6, 2C9, 2C19, or OATP1B3. GS-704277 and GS-441524 metabolites are also not the substrates of CYP2C19 or 3A4, CYP1A1, 1A2, 2B6, 2C8, 2C9, 2D6, or 3A5 (EMA, 2020; Yang, 2020; Deb et al., 2021b; US-FDA, 2022). The present study was undertaken with herbal preparations using human liver microsomes to evaluate their effect on selective CYP enzymes to predict the possible HDIs implying efficacious and safer management of COVID-19.

## Materials and methods

### Chemicals reagents and solvents

Testosterone, ketoconazole, 6β-hydroxy testosterone, paclitaxel, rosiglitazone, remdesivir, NADPH, 6-hydroxy paclitaxel, dextromethorphan HBr, dextrorphan, quinidine, NADPH, and mixed gender HLM (human liver microsomes: M0317; Sigma-Aldrich) were used in this study. Water, methanol, and acetonitrile of LC-MS grade were obtained from Merck Life Science, Pvt. Ltd, India. The aqueous extracts of *Withania somnifera* (Batch No.: 012619D0411WSEP) and AYUSH-64 (Batch No.: 89–1/2020-CCRAS/Admn) were prepared in a GMP-certified facility and were provided by the National Medicinal Plants Board (NMPB), Ministry of AYUSH (Ayurveda, Yoga, Naturopathy, Unani, Siddha, and Homeopathy), Government of India. The aqueous extract of *ashwagandha* was prepared as per the Ayurvedic procedure given in classical texts (Chunekar, 1999; Gogaṭ;e, 2000) and the Ayurvedic Pharmacopoeia of India (Part-I, Vol-I, Pages: 19–20). Briefly, the aqueous extract of *ashwagandha* was prepared from the dried roots and soaked in an extraction vessel with RO water in a 1:4 ratio. It was extracted for 3 h at 60 ± 5°C, and then it was filtered through a 400-micron sieve. The material was subsequently extracted two more times, and collected filtrates were pooled and concentrated. The concentrated mass was dried to obtain a powdered extract. AYUSH-64 is a polyhedral formulation prepared as per Ayurvedic principles; it is an Ayurvedic Proprietary Medicine from the Central Council for Research in Ayurvedic Sciences (CCRAS).

### Phytochemical characterization of *ashwagandha* and AYUSH-64 using LC-MS/MS

The lyophilized aqueous extracts of *ashwagandha* and AYUSH-64 were reconstituted (10 mg/ml) in 100% methanol followed by sonication for 30 min. The sample was filtered through a 0.2-mm filter and analyzed with UHPLC Ultimate 3000 coupled with an Orbitrap mass analyzer. The HSS-T3 C18 column (2.1 × 100 mm, 1.8 µm, 100 Å; Waters Corporation) was used for chromatographic separations, and the column oven temperature was maintained at 40°C. Mobile phases A and B contain water with 0.1% formic acid and acetonitrile with 0.1% formic acid, respectively. The gradient elution started with 1% B to 95% B over 14 min (Kamboj et al., 2021). The Orbitrap Fusion mass spectrometer fitted with heated electrospray ionization (HESI) was operated for positive and negative ion modes at 1,20,000 resolution in the MS1 mode and 30,000 resolution in the data-dependent MS2 scan mode. The spray voltage used for these positive and negative modes is 4,000 and 3,500 V, respectively. Sheath gas and auxiliary gas were set at 42 and 11, respectively. The mass scan range was at 50–1,000 m/z, the AGC (automatic gain control) target was at 200,000 ions and the maximum injection time was 80 ms for MS, and the AGC target was 20,000 ions and the maximum injection time was 60 ms for MS/MS (Kamboj et al., 2021). Data processing was performed using Thermo Scientific Xcalibur software, and the identification of metabolite was confirmed by accurate mass and MS/MS fragmentation match of the metabolites available in the literature and mzCloud database.

### Predicting herb−drug interactions: An *in silico* approach

The list of characterized phytoconstituents was further used for predicting HDIs for safe and effective usage along with drugs used in the management of COVID-19 and associated comorbidities. Therefore, the *in silico* pharmacokinetic studies were explored using the SwissADME tool (Daina et al., 2017) to correlate with *in vitro* studies of herbal formulations. To predict the HDI, the ADME data of these phytoconstituents were used to assess any interaction of COVID-19 drugs with the *in silico* predicted target that are involved in the ADME properties of these phytoconstituents.

### CYP inhibition activity assay

CYP3A4, 2C8, and 2D6 activity inhibition assays were performed by using HLM at a protein concentration of 10 mg/ml. Testosterone (70 µM), paclitaxel (5 µM), and dextromethorphan (5 µM) are used as probe substrates for CYP3A4, 2C8, and 2D6, respectively. The marker metabolites 6β-hydroxy testosterone, 6-hydroxy paclitaxel, and dextrorphan were used for measuring the enzyme activity. The incubation mixture was prepared by gentle mixing of PBS (pH 7.4; 100 mM), HLM (20 mg/ml), and DMSO. The amount of PBS, HLM, and DMSO in the incubation mixture was 98.57%, 1.2%, and 0.23% for CYP3A4 and CYP2D6, while for CYP2C8 it was 98.87%, 0.9%, and 0.23%, respectively. For each isozyme, an aliquot of 99.5 µ of the incubation mixture (containing HLM and the substrate) was spiked with 0.5 µl of investigational herbal drug/positive control/remdesivir working solution in a microcentrifuge tube and mixed by swirling and mild shaking. Thereafter, 10 µl of 10 mM NADPH was added to initiate the reaction and incubated additionally for 10 min at 37°C. The reactions were stopped with the addition of 400 µl of quenching solution (%100 methanol) (Patil et al., 2014). The samples were centrifuged at 10,000 rpm for 5 min, and the supernatants were analyzed using LC-MS/MS. All the experiments were carried out in triplicate. The positive control inhibitors were processed similarly and analyzed concurrently, while the blanks were prepared by spiking DMSO instead of the investigational plant drug/control.

### Preparation of the test solution and positive control

The stock solution (500 mg/ml) of the investigational herbal drugs was prepared in water and kept overnight in a mechanical shaker at a speed of 200 rpm at 37°C. The investigational herbal drugs (*ashwagandha* and AYUSH-64) were prepared in seven different concentrations: 1, 10, 20, 50, 100, 1,000, and 2,000 μg/ml. The solutions were centrifuged at 10,000 rpm for 30 min, and the supernatant collected was used for further analysis. The concentration of positive controls ranged from 0.0005 to 5.00 µM, 1.00 to 0.01 µM, and 5.00 to 0.0005 µM for ketoconazole (CYP3A4) (Patil et al., 2014), rosiglitazone (CYP2C8) (Wu et al., 2014), and quinidine (CYP2D6) (Kerry et al., 1994), respectively. The concentration of case–control small-molecule remdesivir was prepared in a range of 1–1,000 ng/ml (Humeniuk et al., 2020).

### Measurement of marker metabolites by LC-MS/MS

The marker metabolites (6-β-hydroxy testosterone, 6-hydroxy paclitaxel, and dextrorphan) were formed as a result of the metabolism of substrates (testosterone, paclitaxel, and dextromethorphan) by CYP3A4, CYP2D6, and CYP2C8, respectively, and measured using LC-MS/MS. Water with 0.1% formic acid was used as the mobile phase A and methanol and 0.1% formic acid as mobile phase B. The run time of 14 min with a flow rate of 0.300 ml/min was used. The chromatography separation method employed was similar to the conditions mentioned in section 3.2.

### Data processing and targeted metabolite analysis

A Thermo Scientific Xcalibur system was used for data processing and data analysis. The standard metabolite retention time and MS/MS fragmentation were matched with samples. The respective metabolite concentration was monitored accordingly under different experimental conditions (CYP3A4, CYP2D6, and CYP2C8).

### Determination of IC_50_ values

The aforementioned concentrations of investigational herbal drugs (refer to section 3.5) (*ashwagandha* and AYUSH-64) were selected on the basis of the daily maximum human dose when diluted in 1 L of gastrointestinal fluid followed by their distribution in 56 L of total body fluid (Patil et al., 2014). This assumption is based on the Ayurvedic properties of these drugs, thereby considering the one-compartment modeling distribution (Gogte, 2000; Mishra et al., 2010). The percentage (%) control activity and (%) inhibitory activity were calculated using the following formulae (Winslow and Kroll, 1998):

$$\%\;Control\;activity=\frac{Peak\;area\;of\;metabolite\;formed\;in\;the\;presence\;of\;herbs}{Peak\;area\;of\;metabolite\;formed\;in\;control}\;100,$$

% Inhibition activity=100−% Control activity.

### Prediction of probable clinical interactions

IC_50_ of CYP substrate (i.e., testosterone, paclitaxel, and dextromethorphan for CYP3A4, CYP2C8, and CYP2D6, respectively) activity in HLM was calculated graphically by the nonlinear regression analysis of logarithmic inhibitor concentration (log conc.) versus % of the inhibitory activity plot using GraphPad Prism 5. The data were expressed as mean ± standard deviation. The ratio of I/K_i_ was used to predict plausible clinical interactions, and >0.1 was considered for the possible clinical interaction. [I] is the mean maximum surrogate plasma concentration (C_max_) at the steady state after administration of the highest clinical dose of inhibitor in humans as per published reports. The inhibitory constant (K_i_) was calculated using the following equation:

$$Ki=\frac{IC50}{1}+\frac{\left[S\right]}{Km},$$

where [S] and [K_m_] are substrate concentration and Michaelis constant, respectively (Patil et al., 2014). The interactions between ketoconazole and testosterone, rosiglitazone and paclitaxel, and quinidine and dextromethorphan are competitive in nature. In the present study, [S] and [K_m_] values were kept the same. [I] values for ketoconazole were set at 1–5 μg/ml, and likewise, 100 μg/ml and 5 μg/ml were set for rosiglitazone and quinidine, respectively. It was not possible to calculate the I/K_i_ ratio for herbal extracts because of the absence of whole extract pharmacokinetics data (Haupt et al., 2015). Instead, it was specified that the IC_50_ values of whole plant extracts <100 μg/ml and the IC_50_ values of individual plant phytoconstituents <100 μM/ml were considered potent inhibitors of CYP-450 enzymes, which might result in undesirable HDIs (Patil et al., 2014; Borse S. P. and Kamble B. B., 2015; Zhang et al., 2021). The rationale for the same is based on the following:

a) Ayurvedic properties (pharmaceutical and physiological) correlated to the compartmental analysis. Briefly, the Ayurvedic drug properties (*rasa, guna, virya, vipaka,* and *prabhava*) are helpful in deciding to use the test drugs and their putative PK-PD approaches [Gogte, 2000; Mishra et al., 2010; Gundeti et al., 2020a; Ayurvedic Pharmacopoeia of India (Part-I, Vol-I, Pages: 19–20)]. Both the test drugs might follow the BCS Class-I nature of one-compartment distribution (Patil et al., 2014), and it is expected to achieve less than <100 μg/ml concentration for the extracts and <100 μM/ml for the individual plant phytoconstituents. It has been verified in *in vivo* experiments (Patil et al., 2013; Modi et al., 2022). Therefore, the K_i_ value can be calculated with the aforementioned formula.
b) It is further supported by the recent publication on the reliability of estimating K_i_ values for inhibition of CYP from corresponding IC_50_ values (Haupt et al., 2015). It was found that the values of K_i_ and IC_50_ were determined under the following conditions: 1) the concentration of CYP-450 marker substrate [S] equals to K_m_ (for IC_50_ determinations) and spanned K_m_ (for K_i_ determinations); 2) the substrate incubated for a short time (5 min) to minimize metabolism-dependent inhibition and inhibitor depletion; and 3) the concentration of HLM was low (0.1 mg/ml or less) to maximize the unbound fraction of the inhibitor. Under these conditions, predicted K_i_ values, based on IC_50_/2, correlated with the experimentally determined K_i_ (Haupt et al., 2015).

## Results

### Phytochemical characterization of *ashwagandha* and AYUSH-64 using LC-MS/MS

**FIGURE 1:**
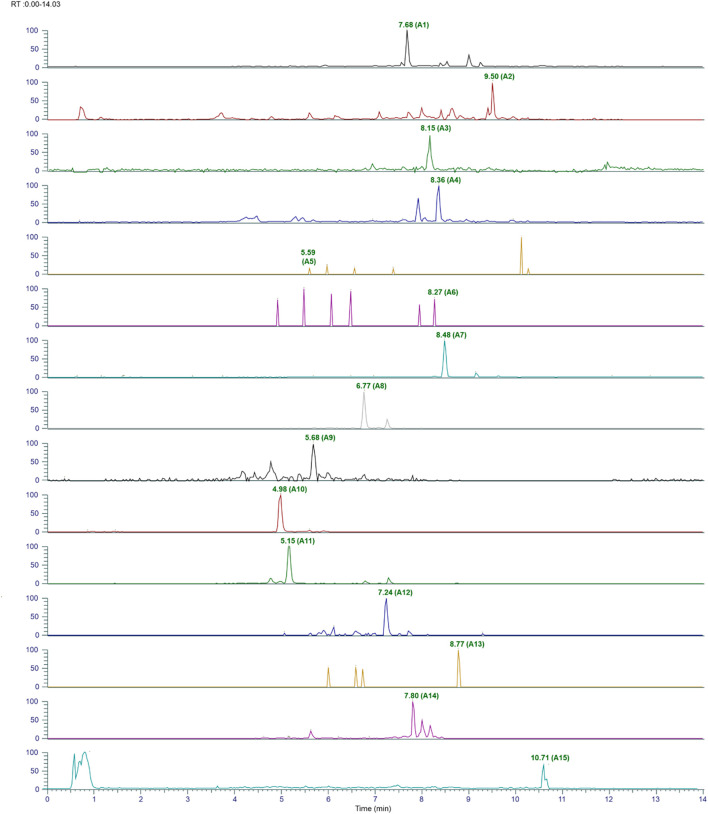
Chromatograms of identified phytoconstituents of AYUSH-64 in the positive ion mode. Betulin (A1), neocaesalpin B (A2), formononetin (A3), burnamine (A4), picrinine (A5), vallesamine (A6), oleanolic acid (A7), amarogentin (A8), gentianine (A9), mangiferin (A10), and sweroside (A11) and (1b) comprising A (12–24); kaempferol (A12), 5-methoxystrictamine (A13), neocaesalpin L (A14), and beta-caesalpin (A15).

**FIGURE 2:**
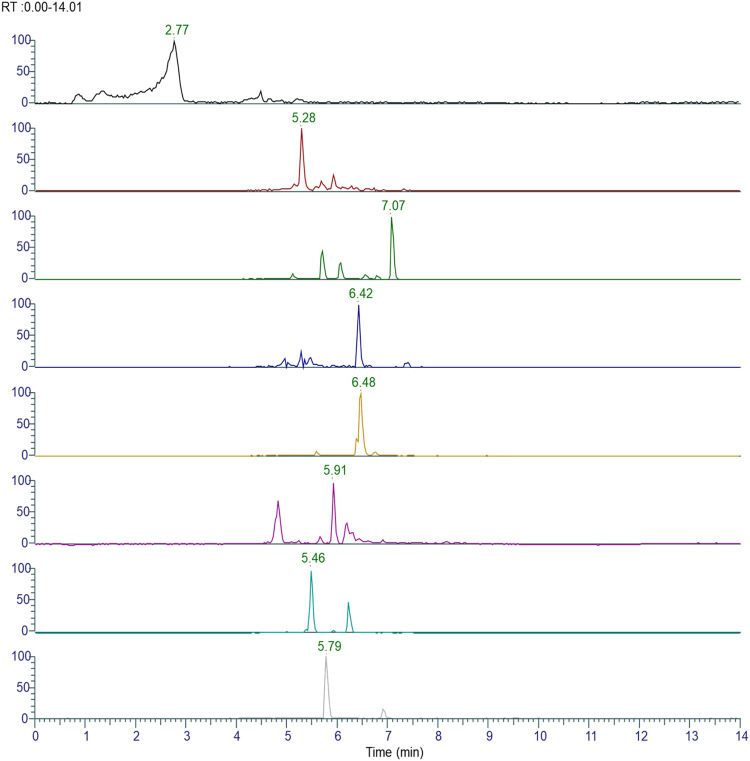
Chromatograms of the identified phytoconstituents of AYUSH-64 in the positive ion mode. Gallic acid (A16), picroside B (A17), verminoside (A18), swertianolin (A19), minecoside (A20), apocynin (A21), picroside-IV (A22), kutkoside (A23), and picroside-II (A24).

**FIGURE 3:**
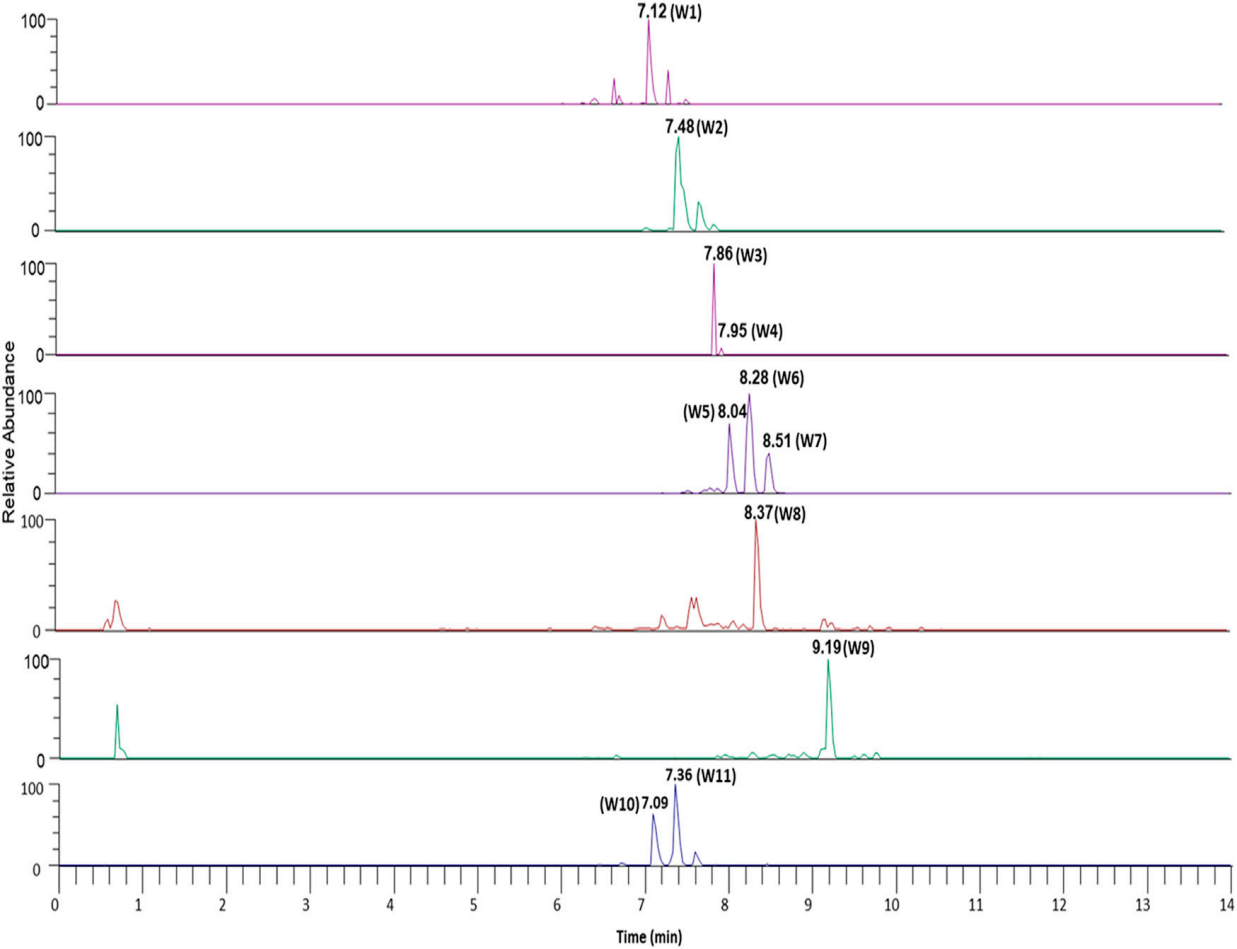
Chromatograms of identified phytoconstituents of W*ithania somnifera* comprising W (1–11); withanoside VII (W1), viscosalactone B (W2), dihydro withaferin A (W3), withanoside V (W4), withaferin A (W5), 12-deoxywithastramonolide (W6), withanolide A (W7), 27-hydroxywithanone (W8), withanolide B (W9), withanoside X (W10), and coagulin Q (W11).

The plant phytoconstituents were characterized by accurate mass and MS/MS fragmentation pattern match. Briefly, 11 and 24 plant phytoconstituents were tentatively identified from the aqueous extracts of *ashwagandha* and AYUSH-64 (Figures 1–3; S2 file). The certificate of analyses of both the aqueous extracts showed heavy metal impurities and microbial load within the acceptable limits and was as per the Indian pharmacopeial standards.

### Prediction of herb−drug interaction studies: an *in silico* approach

**FIGURE 4:**
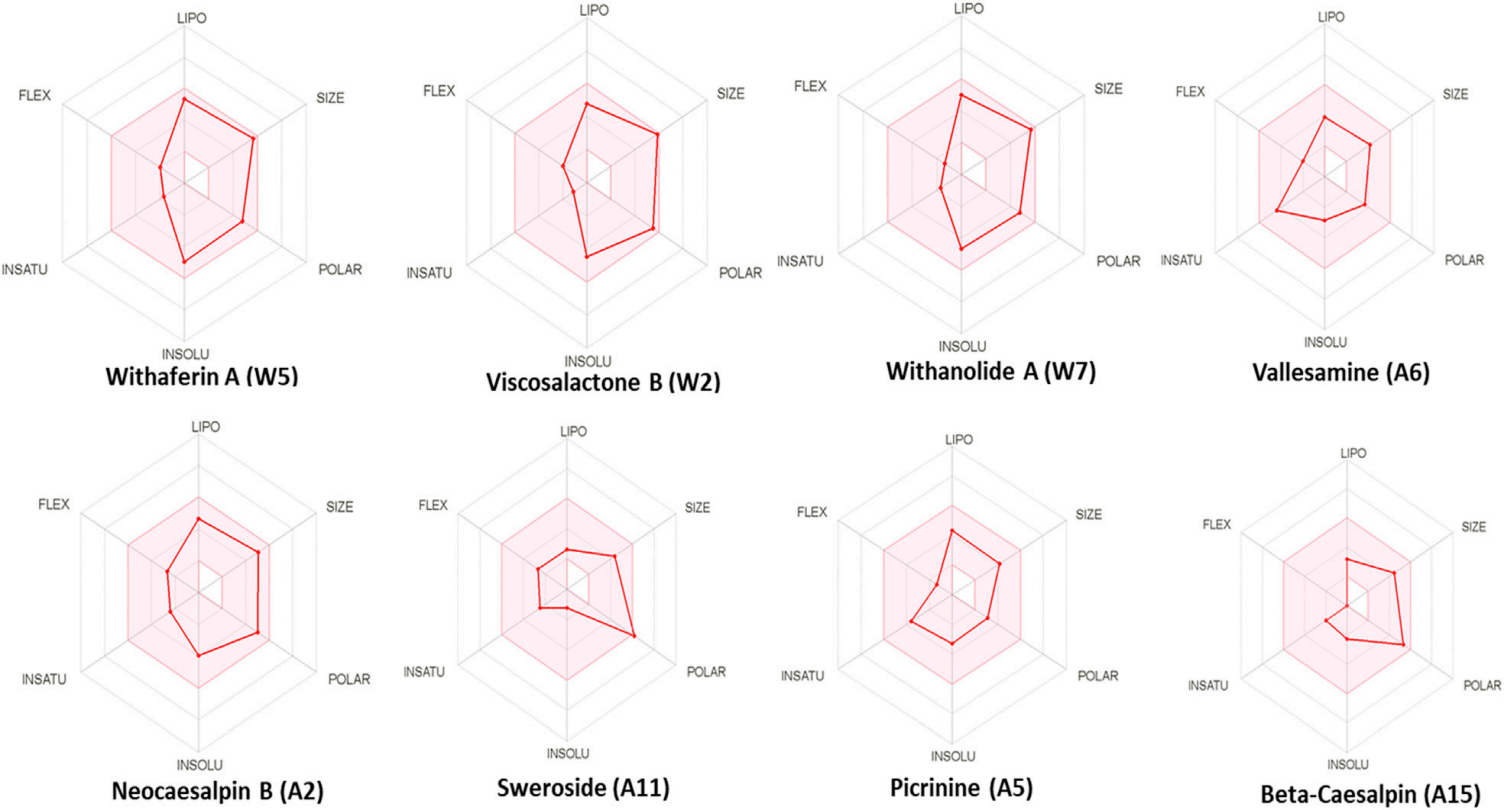
Radar plots of key phytoconstituents present in AYUSH-64 and *Withania somnifera* extracts.

Previously published data on *ashwagandha* and its effect on CYPs (*in silico* and *in vitro*) have shown that the aqueous extracts prepared as per the procedure given in Ayurvedic texts did not show inhibitory effects (Patil et al., 2014; Borse S. and Kamble B., 2015; Borse et al., 2021), whereas, the *in silico* approach was employed to predict the ADME properties of the herbal extracts. The radar plots of phytoconstituents of AYUSH-64 showed good oral bioavailability of most of the compounds along with good drug likeliness and gastrointestinal (GI) absorption (Figure 4; S1 File). This is in line with Ayurvedic understandings of drug properties. In addition, few of the phytoconstituents of AYUSH-64 showed CYP3A4 inhibitory activity followed by inhibition of other CYP enzymes (CYP2D6, 2C8, 2C19, and 2C9) to a lesser extent. This might be helpful in predicting drug−drug interactions and selection and development of safer dosage regimens, especially in the case of isolated phyto-molecules.

### Activity of CYP isoenzymes in the presence of herbal extracts, remdesivir, and their known inhibitors

**FIGURE 5:**
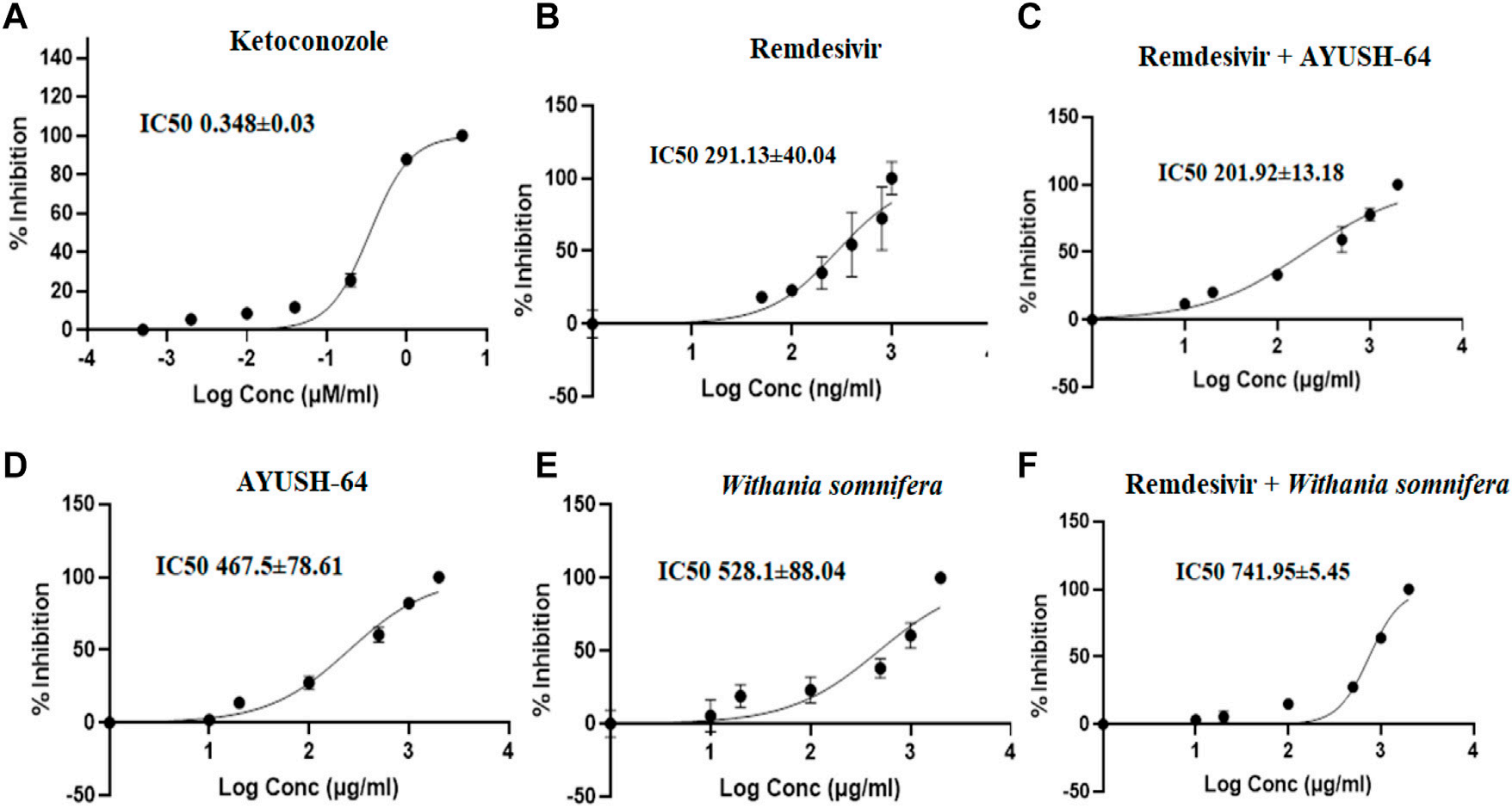
Effect of **(A)** ketoconazole (5.00–0.0005 μM/ml), **(B)** remdesivir (1,000–1 ng/ml), **(C)** remdesivir + AYUSH-64 (2001–1.001 μg/ml), **(D)** AYUSH-64 (2000–1 μg/ml), **(E)** *Withania somnifera* (2000–1 μg/ml), and **(F)** remdesivir + *Withania somnifera* (2001–1.001 μg/ml) on CYP3A4-mediated hydroxylation of testosterone. The graphs have been plotted for log conc. *vs*. percentage of inhibition.

The effect of aqueous extracts of *ashwagandha*, AYUSH-64, and remdesivir was investigated on CYP3A4, 2C8, and 2D6 by using HLM. The respective marker metabolites from each enzymatic reaction (CYP3A4, 2D6, and 2C8) were quantified (S1 File). In the present study, the I/K_i_ ratio of ketoconazole (CYP3A4 inhibitor) was 28.73, while for remdesivir it was 6.87, which showed moderate/weak inhibition of CYP3A4 as compared to ketoconazole. The results were consistent with the previous reports (Table 2, Figure 5). On the other hand, *ashwagandha* or AYUSH-64 alone and in combination with remdesivir did not show CYP3A4 inhibition as their respective IC_50_ value was >100 μg/ml (Table 1, Figure 5).

**TABLE 1:** IC_50_ values of *Withania somnifera* and AYUSH-64 extracts for CYP3A4, 2C8, and 2D6 isoenzymes.

Compound name	CYP3A4	CYP2C8	CYP2D6
	IC_50_ (µg/ml)	IC_50_ (µg/ml)	IC_50_ (µg/ml)
*Withania somnifera*	528.1 ± 88.04	233.62 ± 36.72	161.9 ± 4.45
AYUSH-64	467.5 ± 78.61	63.44 ± 14.9	216.25 ± 39.5
Remdesivir + *Withania somnifera*	741.95 ± 5.45	116.39 ± 39.2	108.68 ± 23.94
Remdesivir + AYUSH-64	201.92 ± 13.18	85.21 ± 18.3	111.05 ± 5.07

**FIGURE 6:**
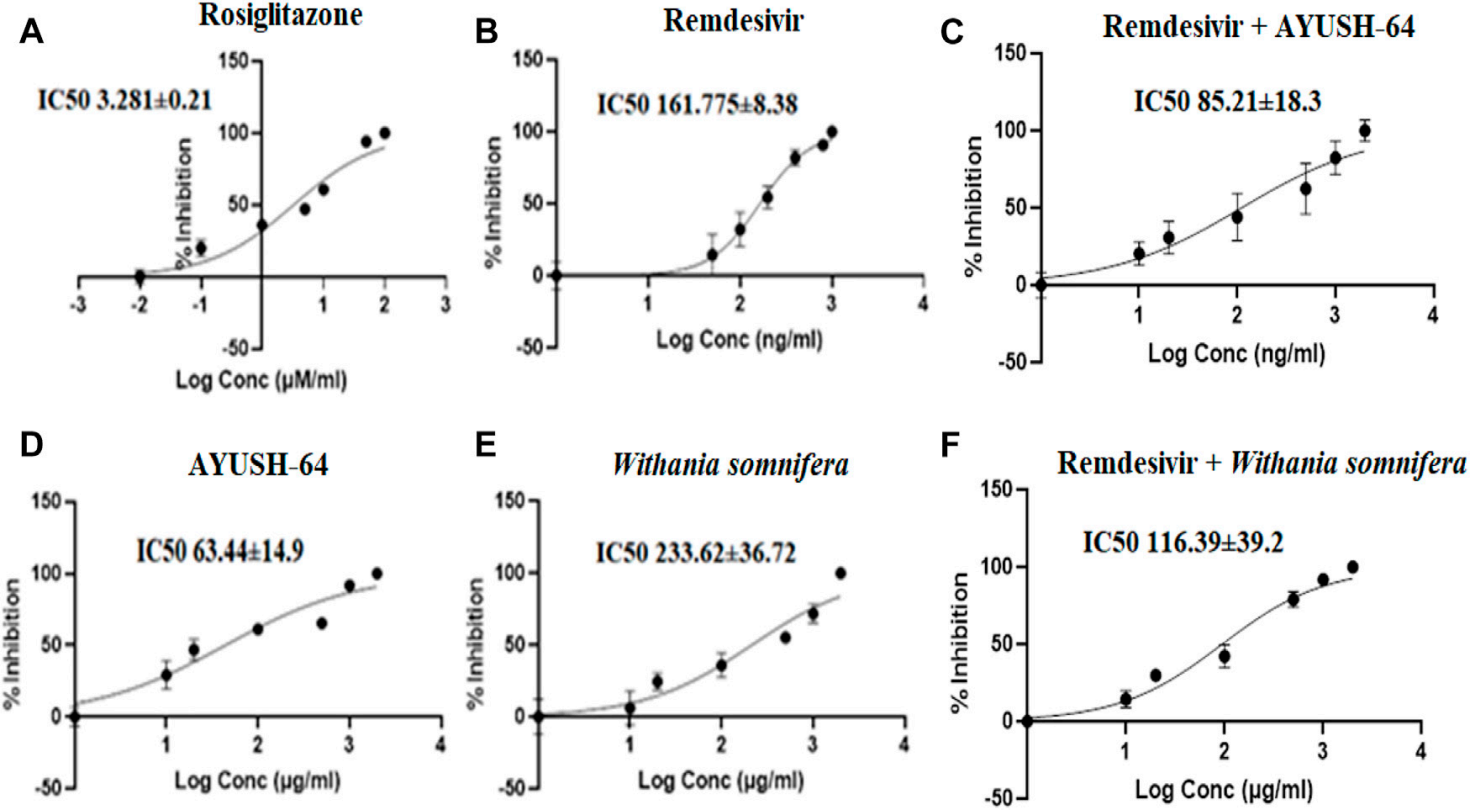
Effect of **(A)** rosiglitazone (100–0.01 μM/ml), **(B)** remdesivir (1,000–1 ng/ml), **(C)** remdesivir + AYUSH-64 (2001–1.001 μg/ml), **(D)** AYUSH-64 (2000–1 μg/ml), **(E)** *Withania somnifera* (2000–1 μg/ml), and **(F)** remdesivir + *Withania somnifera* (2001–1.001 μg/ml) on CYP2C8-mediated hydroxylation of paclitaxel. The graphs have been plotted for log conc. *vs*. percentage of inhibition.

Similarly, the I/K_i_ ratio of rosiglitazone, a CYP2C8 inhibitor, was 60.97, while the I/K_i_ ratio of remdesivir was 12.36, suggesting a weak inhibition of CYP2C8 as compared to rosiglitazone (Table 2, Figure 6). Similarly, *ashwagandha* alone and in combination with remdesivir did not exhibit any inhibitory effect (IC_50_ > 100 μg/ml) on the CYP2C8 isoenzyme system, while AYUSH-64 displayed moderate to weak inhibition as IC_50_ was <100 μg/ml. In addition, AYUSH-64 in combination with remdesivir (IC_50_ = 85.21 ± 18.3) also showed a similar trend (Table 1, Figure 6).

**TABLE 2:** IC_50_ values of standard CYP substrate inhibitors and remdesivir for CYP3A4, 2C8, and 2D6 isoenzymes.

CYP3A4				CYP2C8				CYP2D6			
Compound name	IC_50_	K_i_	I/K_i_	Compound name	IC_50_	K_i_	I/K_i_	Compound name	IC_50_	K_i_	I/K_i_
Ketoconazole (µM/ml)	0.348 ± 0.03	0.17	28.73	Rosiglitazone (µM/ml)	3.28 ± 0.21	1.6	60.9	Quinidine (µM/ml)	0.12 ±	0.06	83.3
									0.001		
Remdesivir (ng/ml)	291.13 ± 40.04	145.5	6.87	Remdesivir (ng/ml)	161.77 ± 8.38	80.8	12.3	Remdesivir (ng/ml)	327.25 ± 79.5	163.6	6.1

**FIGURE 7:**
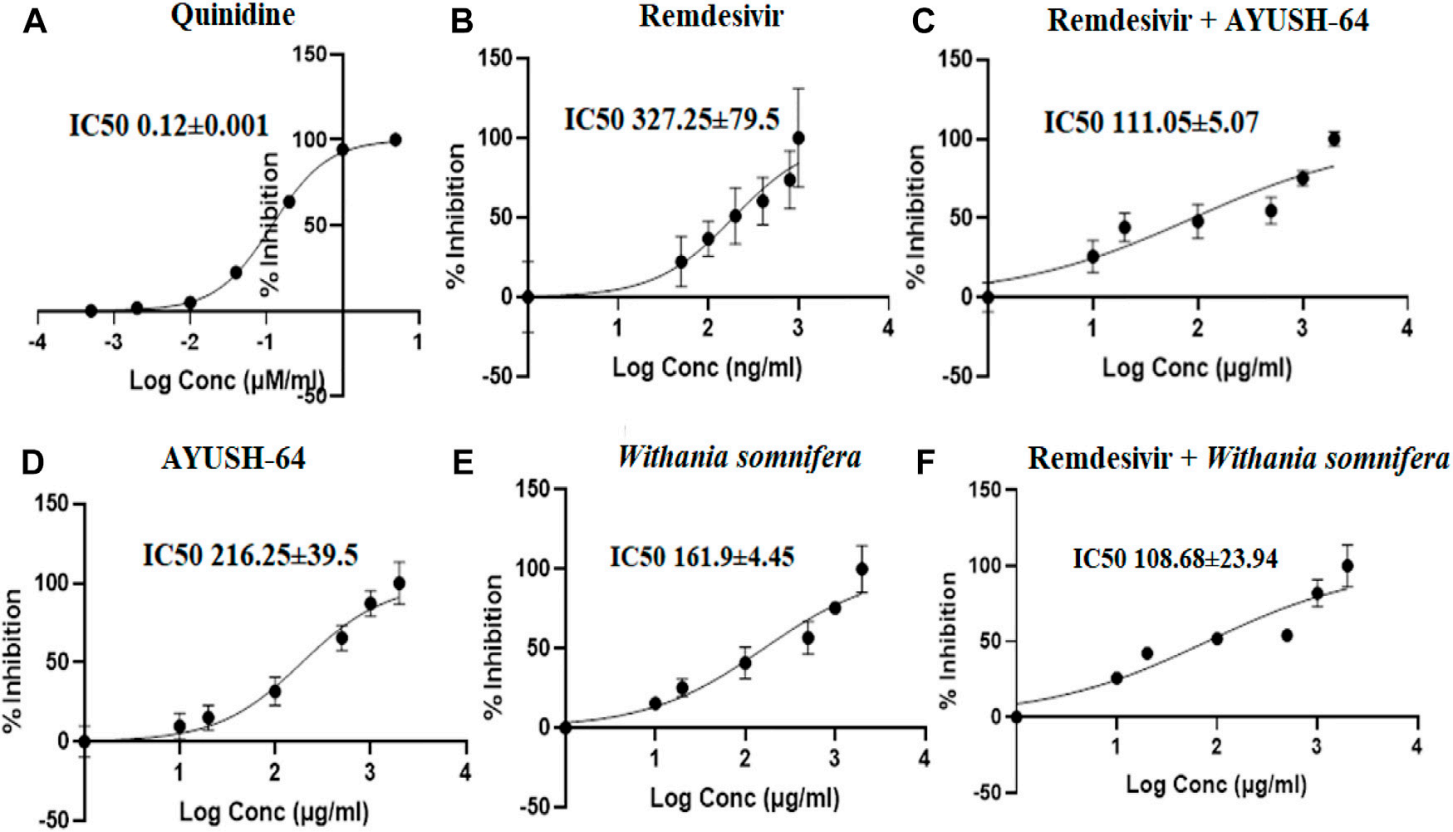
Effect of **(A)** quinidine (100–0.01 μM/ml), **(B)** remdesivir (1,000–1 ng/ml), **(C)** remdesivir + AYUSH-64 (2001–1.001 μg/ml), **(D)** AYUSH-64 (2000–1 μg/ml), **(E)** *Withania somnifera* (2000–1 μg/ml), and **(F)** remdesivir + *Withania somnifera* (2001–1.001 μg/ml) on CYP2D6-mediated hydroxylation of dextromethorphan. The graphs have been plotted for log conc. *vs.* percentage of inhibition.

The I/K_i_ ratio of quinidine, a CYP2D6 inhibitor, and remdesivir was 83.3 and 6.1, respectively (Table 2, Figure 7). The experiment thus suggests a weak inhibition of CYP2D6 by remdesivir as compared to quinidine. In addition, *ashwagandha* and AYUSH-64 alone and in combination with remdesivir did not exert any inhibitory effect on CYP2D6 isoenzyme, as the IC_50_ value was >100 μg/ml (Table 1, Figure 7).

## Discussion

Currently, several drugs and therapies are in use to mitigate or prevent illnesses caused by COVID-19, even though strategies for new drug discovery and repurposing of existing drugs are under evaluation (Mei and Tan, 2021; Parasher, 2021). The use of integrative medicine (IM) is an attractive option either as a primary (to treat a disease/illness) or secondary (to downgrade the prevalence of a disease/illness) prevention (Sofowora et al., 2013). Moreover, the ideal therapeutic regime to treat COVID-19 is expected to possess diverse activities such as immunomodulatory, adaptogenic, rejuvenating, anti-stress, anti-inflammatory, and anti-viral activity (Chaturvedi et al., 2020; Borse et al., 2021). It is important that IM should be safe and efficacious to prevent or control the associated comorbidities (Zhang et al., 2022). On the basis of the existing literature, the identified phytoconstituents are known to possess immunomodulatory and anti-viral properties, suggesting their potential use as an adjuvant for COVID-19 therapy (Mani et al., 2020). The herbal extracts/formulations are a mixture of a large number of phytoconstituents rendering them to have multiple pharmacological activities, which might also have untoward interactions if administered along with the conventional medicines (herb−drug interactions) (Izzo and Ernst, 2009; Ye et al., 2021b). HDIs can thus be beneficial, harmful, or even fatal. *Ashwagandha*, a well-known *Rasayana* (∼rejuvenator) also referred to as “Indian ginseng,” is well established for its immunomodulatory, adaptogenic, anti-cancer, anti-diabetic, and anti-COVID-19 activities (Gogte, 2000).

In the present study, we first characterized the aqueous extract of *ashwagandha* and putatively identified 11 important withanolides and withanoside glycosides based on their MS/MS spectra. These glycosides like withanoside VII, V, and X are known to elicit immunomodulatory and anti-viral activities (Girme et al., 2020). On the basis of the existing literature, the identified phytoconstituents are known to possess immunomodulatory and anti-viral properties, suggesting their potential use as an adjuvant for COVID-19 therapy (Mani et al., 2020). Similarly, the aqueous extract of AYUSH-64, a polyherbal formulation, was also characterized using MS/MS fragmentation pattern, and 24 plant phytoconstituents have been putatively identified.

The *in silico* pharmacokinetics of *ashwagandha* and AYUSH-64 revealed that most of the key phytoconstituents of *ashwagandha* and AYUSH-64 seem to have good oral bioavailability, drug-like properties, and GI affinity. In addition, bioinformatics studies from the existing literature revealed that withaferin A, viscosalactone B, withanolide A, vallesamine, neocaesalpin B, sweroside, picrinine, and β-caesalpin have good docking scores and may possess anti-viral activity against SARS-CoV-2 suggesting their potential use in the treatment of COVID-19 (Gundeti et al., 2020b; Kar et al., 2020; Ram et al., 2021; Saggam et al., 2021). Moreover, our *in silico* pharmacokinetic studies indicated that some of the bioactive compounds might have an inhibitory affinity toward cytochrome P450 enzymes (CYP3A4, 2C8, 2C9, 2D6, and 1A2). However, *in vitro* experiments with human liver microsomes exhibited IC_50_ values above 100 μg/ml, rendering that the extract is safe for use. The present study thus predicts the safety of the tested herbal extracts and their use with other conventional drugs for the management of COVID-19. We, however, did not examine the time-dependent change in the CYP activity, and thus we do not completely rule out their interaction with CYP protein or modulation in their expression. Future *in vitro* studies with individual phytoconstituents, *in vivo studies,* and clinical HDI studies might therefore be helpful in assessing their safety.

Such studies are needed to promote the use of herbal medicines alone and/or in combination with conventional drugs (Winslow and Kroll, 1998). Hence, the impact of herbal extracts and their individual phytoconstituents on cytochrome P450 enzymes should be properly investigated to predict their plausible metabolic interactions (Ekor, 2014). In the present study, we also evaluated the effect of the extracts in combination with remdesivir as a case example to understand their interaction with common CYP isoenzymes (Eastman et al., 2020). The study thus focused on CYP enzymes that metabolize 60–80% of the xenobiotic spectrum. CYP3A4 alone is responsible for the metabolism of more than 50% of all xenobiotics prescribed during various illnesses (Zhou et al., 2007), whereas CYP2C8 and CYP2D6 are responsible for 5% and 20% of xenobiotic metabolism, respectively (Desta and Flockhart, 2017; Tirona and Kim, 2017). Remdesivir is known to be the substrate of CYP3A4, 2D6, and 2C8 (Yang, 2020). The results from this study indicated that the [I]/K_i_ ratio of remdesivir for CYP3A4, 2D6, and 2C8 was 38.07, 27.04, and 16.62, respectively, which is similar to the existing reports (EMA, 2020; Deb et al., 2021a). However, the aqueous extract of *ashwagandha* did not show any inhibitory potential, as the corresponding IC_50_ value was >100 μg/ml for all three CYP isoenzymes (Savai et al., 2015). Patil *et. al.* and others also demonstrated that aqueous extracts prepared as per the Ayurvedic procedure did not inhibit CYP3A4 (Patil et al., 2014; Borse S. and Kamble B., 2015; Borse et al., 2021). In addition, *ashwagandha* in combination with remdesivir exhibited IC_50_ > 100 μg/ml, thus indicating a weak inhibitory potential of remdesivir in the presence of the aqueous extract of *ashwagandha*. The combination of *Withania somnifera* and remdesivir or any other drug which is the substrate of CYP3A4, 2D6, and 2C8 seems to be safe for pharmacotherapy.

Furthermore, we also studied the HDIs of polyherbal formulation (AYUSH-64) alone and in combination with remdesivir. The results obtained signify that AYUSH-64 had no inhibitory interaction with CYP3A4 and CYP2D6 as IC_50_ values were found to be > 100 μg/ml, while it had a weak or moderate inhibitory interaction with CYP2C8 with an IC_50_ value of 63.44 ± 14.9. It is therefore inferred that AYUSH-64 seems to be safe to use along with the substrates of CYP3A4 and 2D6, while a caution is warranted for its use with drugs that are substrates of CYP2C8. Moreover, the action of AYUSH-64 in combination with remdesivir showed no inhibitory kind of interaction toward CYP3A4 and 2D6 (IC_50_ value > 100 μg/ml). Thus, both AYUSH-64 and remdesivir had weak or moderate interaction with CYP2C8, and the IC_50_ of the combination was 85.21 ± 18.3. Indeed, it is possible that these two together might compete with each other and exhibit moderate inhibition of CYP2C8 activity. Interestingly, there was no mention of any significant interaction or adverse reaction during the clinical studies conducted with *ashwagandha* and AYUSH-64 (Gundeti et al., 2020a; Reddy et al., 2020; Chopra et al., 2021; Chopra et al., 2022; Singh et al., 2022). The HDIs and pharmacokinetic parameters presented in the current study provide insights into having safer therapeutic options for mitigating SARS-CoV-2 as well as their use for the treatment of chronic diseases.

## Conclusion

Overall, the use of Ayurvedic formulations as an adjuvant with conventional therapy seems to have additional benefits in the management of chronic diseases and COVID-19. AYUSH-64 and *ashwagandha* have been in human use for a long period, and so far, no serious adverse effects have been recorded. The bioactive compounds of *ashwagandha* and AYUSH-64 are known to elicit immunomodulatory activity and possess plausible anti-viral properties. However, to accrue the potential benefit of using the integrative approach for managing complex diseases, it is pertinent to have a proper scientific assessment of the herb−drug interactions. Limited *in vitro* CYP inhibition studies in the present investigation with *ashwagandha* and remdesivir demonstrated probable safety of the combination. Moreover, AYUSH-64 did not exhibit an inhibitory effect on CYP3A4 and 2D6, while a moderate inhibition of CYP2C8 was observed, warranting a degree of caution for integrative pharmacotherapeutic management. Well-designed experimental and clinical studies would be more helpful to further demonstrate their safety.

## Data Availability

The original contributions presented in the study are included in the article/Supplementary Material; further inquiries can be directed to the corresponding authors.
