# Supplementary material for: In vitro effect of Withania somnifera, AYUSH-64, and remdesivir on the activity of CYP-450 enzymes: Implications for possible herb−drug interactions in the management of COVID-19
Source: Front Pharmacol. 2022 Oct 12;13:973768. doi: 10.3389/fphar.2022.973768 (PMC9597875; doi:10.3389/fphar.2022.973768)

## Slide 1
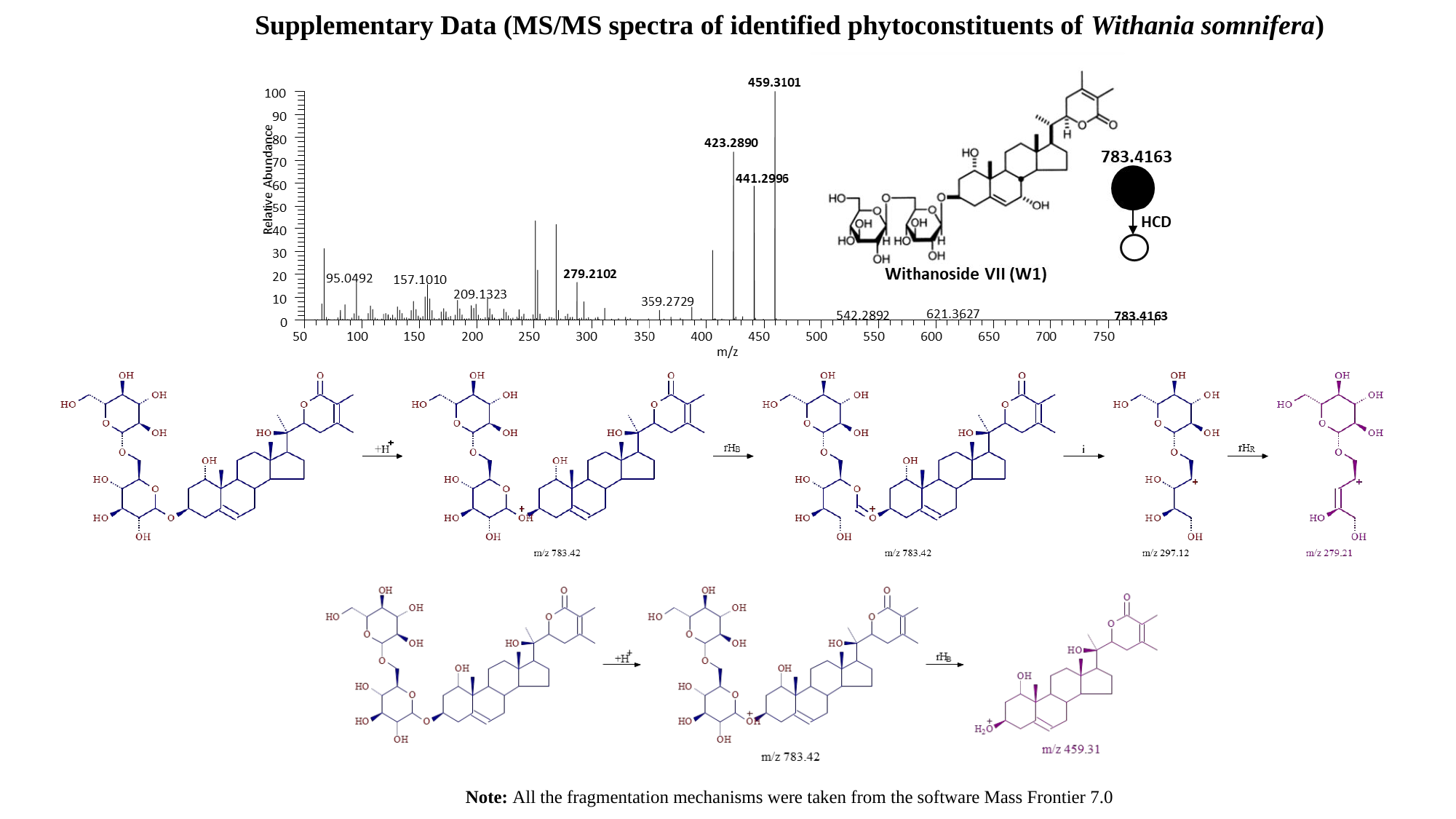

Supplementary Data (MS/MS spectra of identified phytoconstituents of Withania somnifera)
Note: All the fragmentation mechanisms were taken from the software Mass Frontier 7.0

## Slide 2
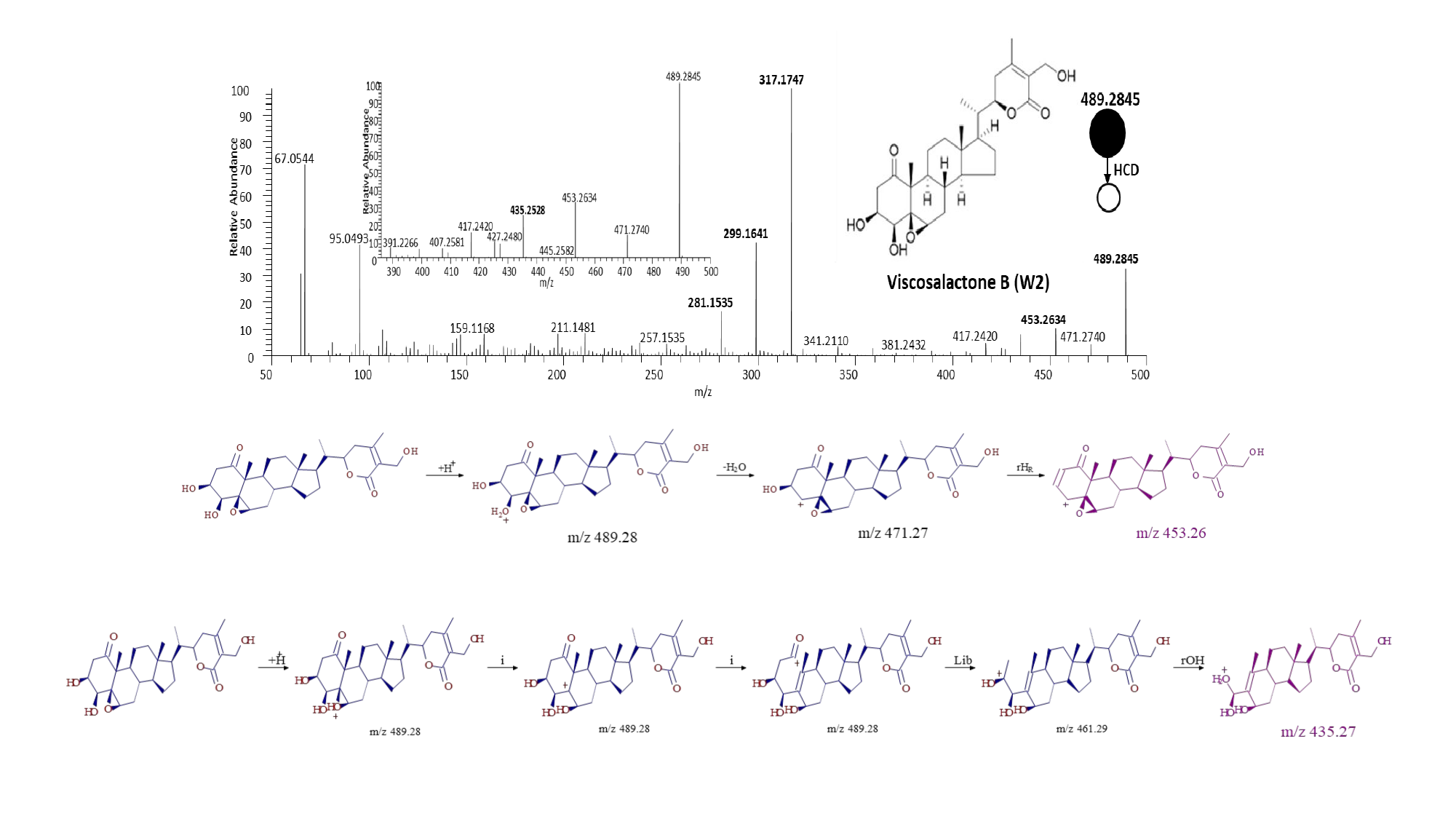

## Slide 3
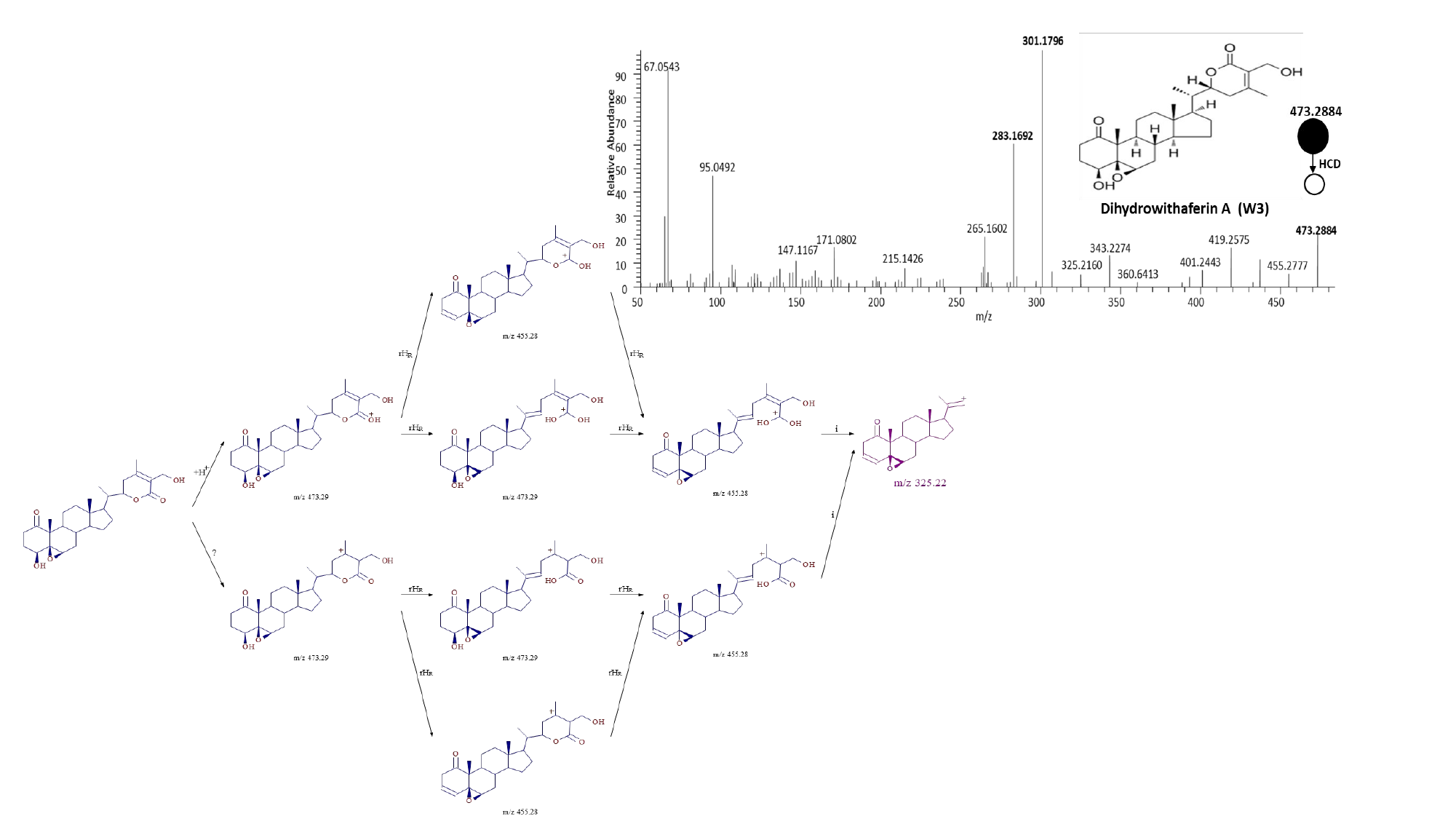

## Slide 4
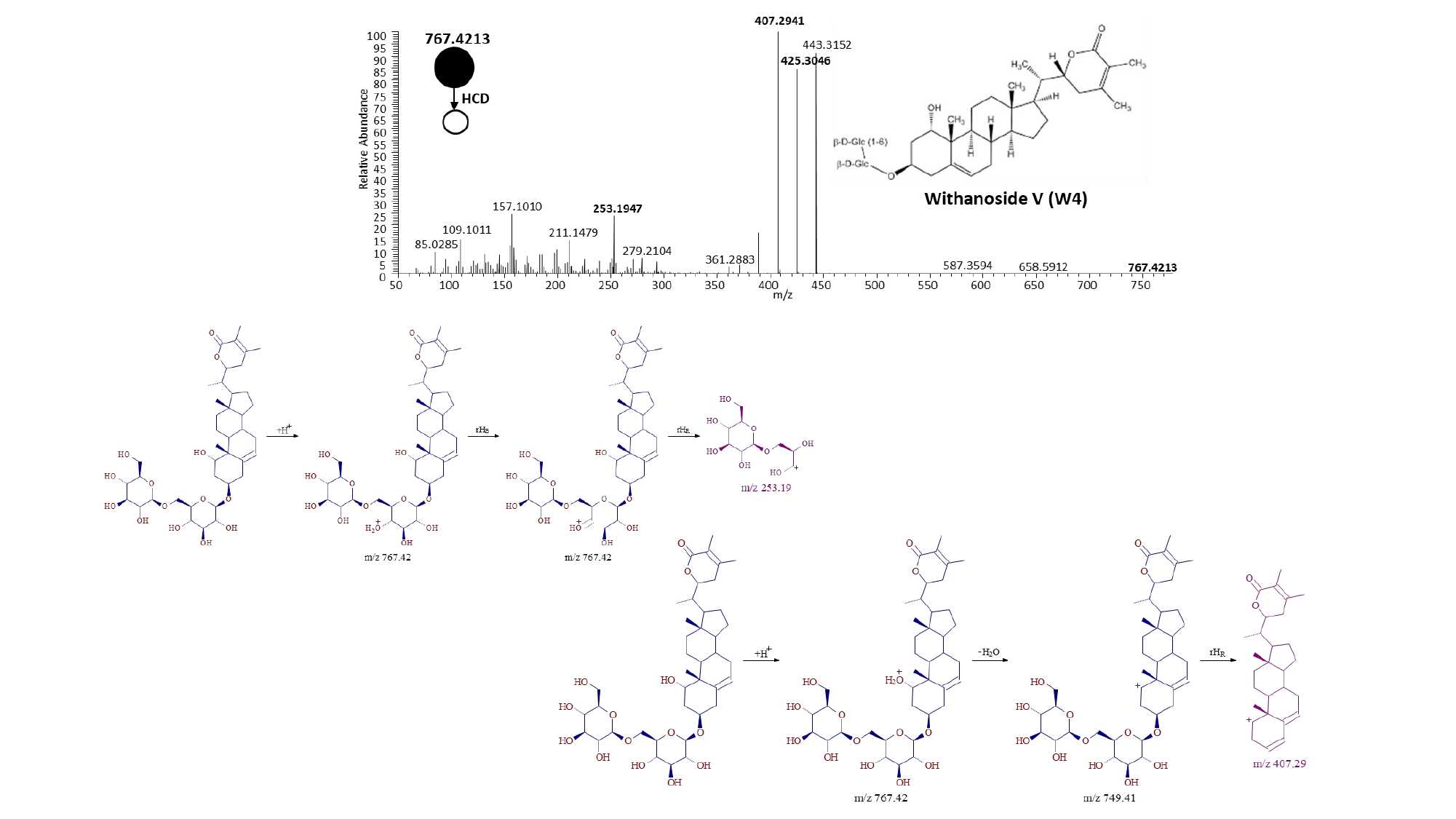

## Slide 5
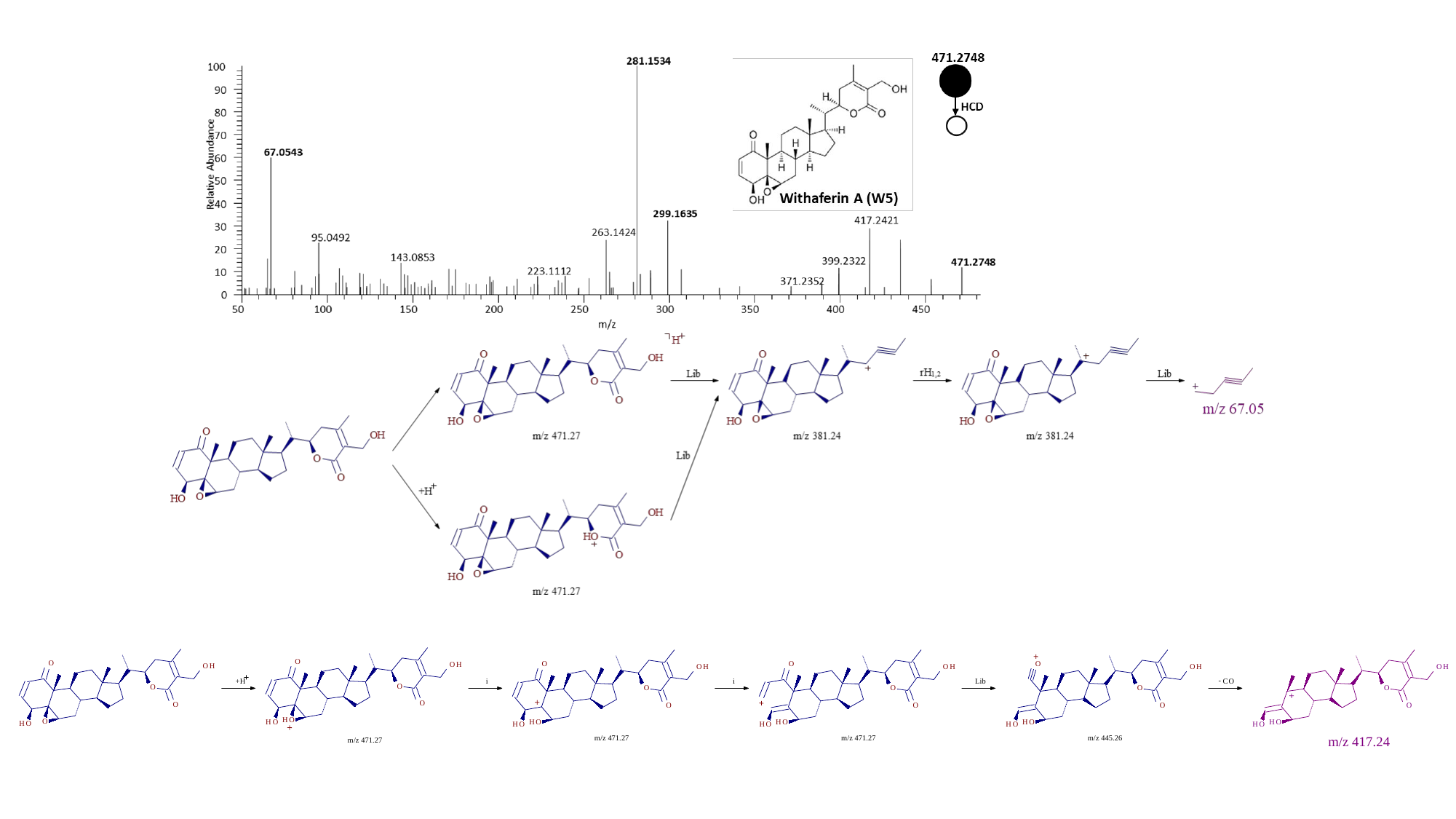

O
O
O
O
H
O
H
O
H
+H
i
O
O
O
O
O
O
H
O
O
H
O
H
O
H
O
H
O
m/z 471.27
m/z 471.27
O
O
O
H
O
H
O
H
-
i
Lib
C
O
O
O
O
O
O
O
H
O
H
O
H
O
H
O
H
O
H
O
m/z 471.27
m/z 445.26
m/z 417.24

## Slide 6
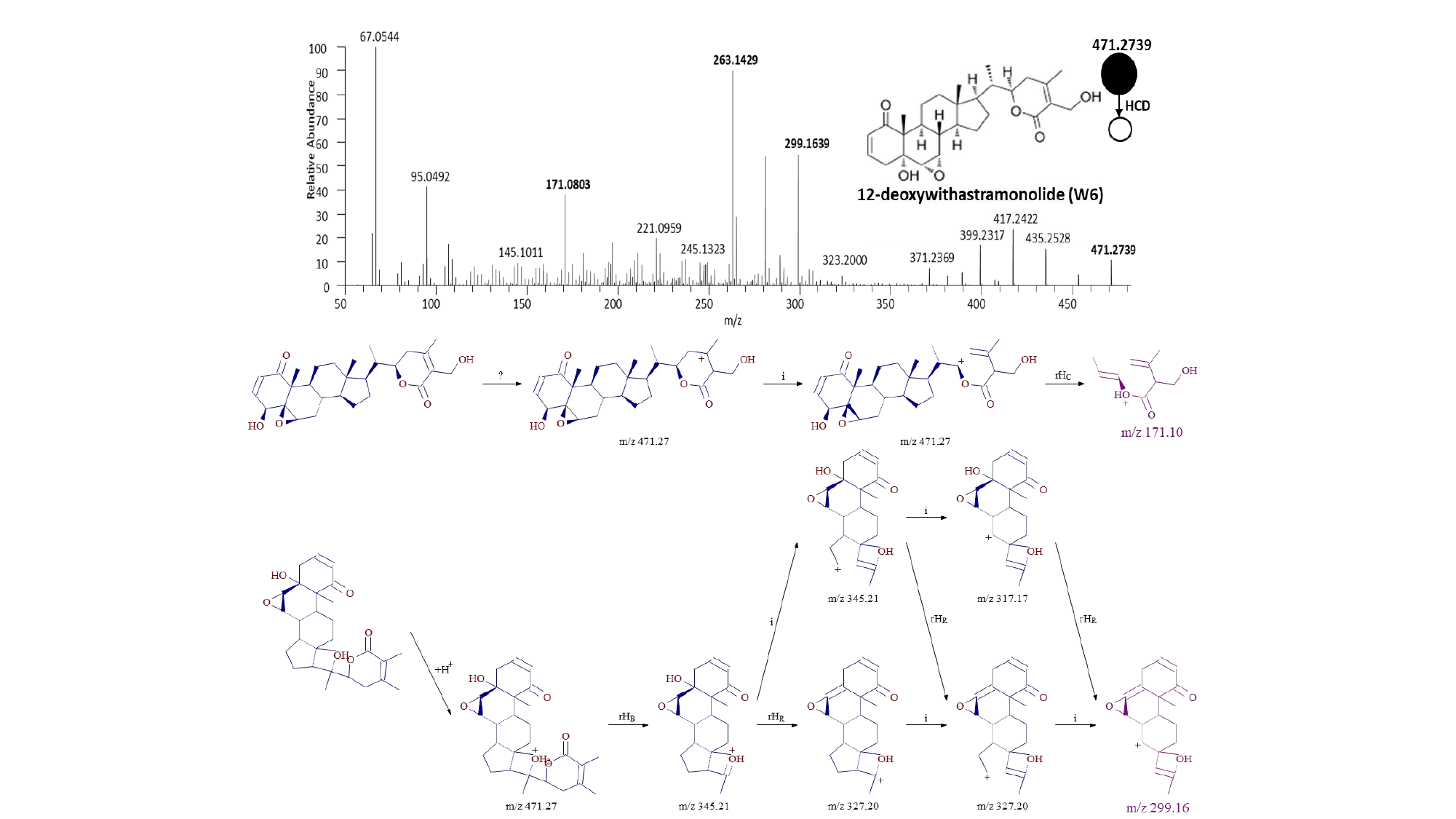

## Slide 7
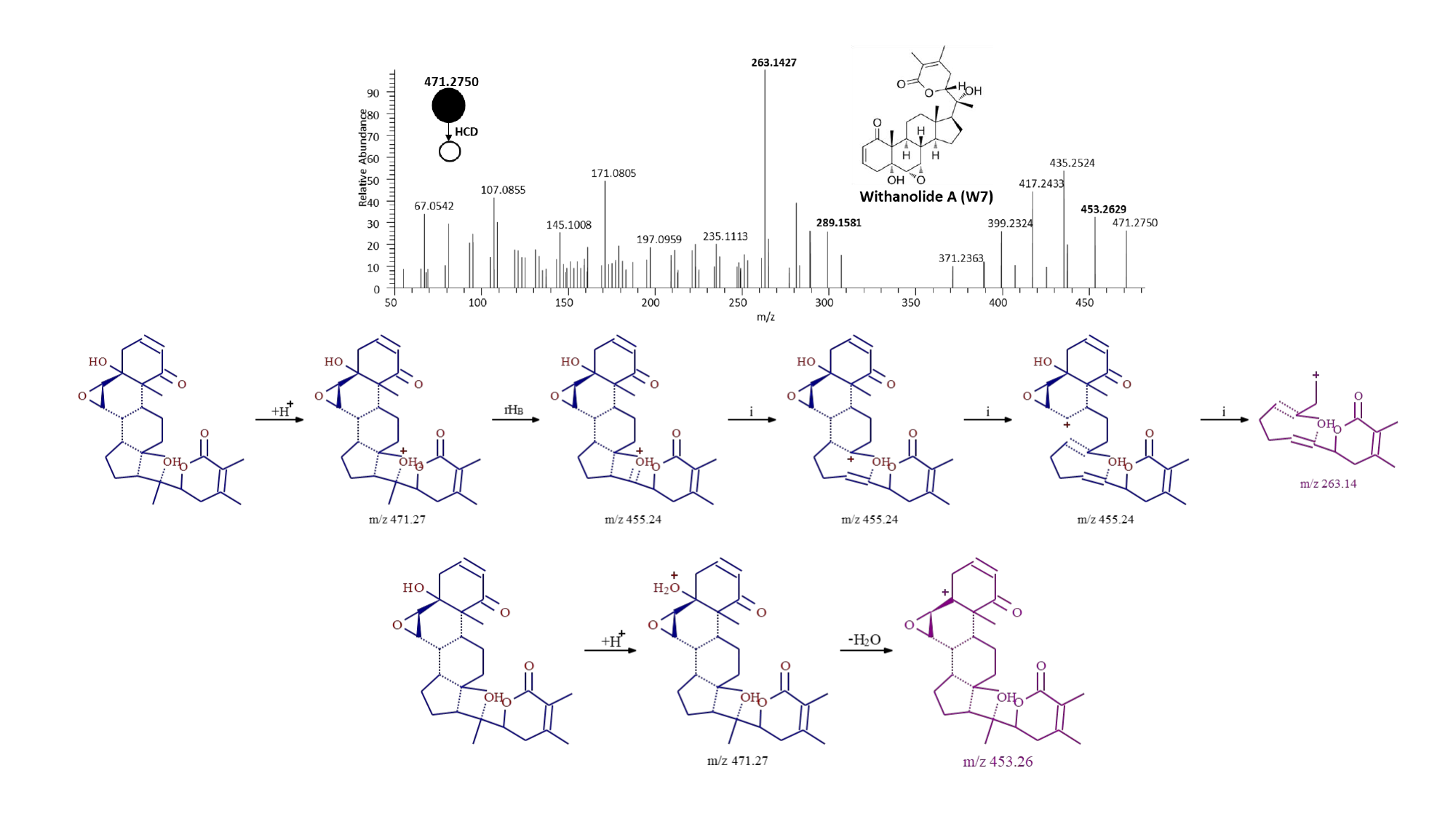

## Slide 8
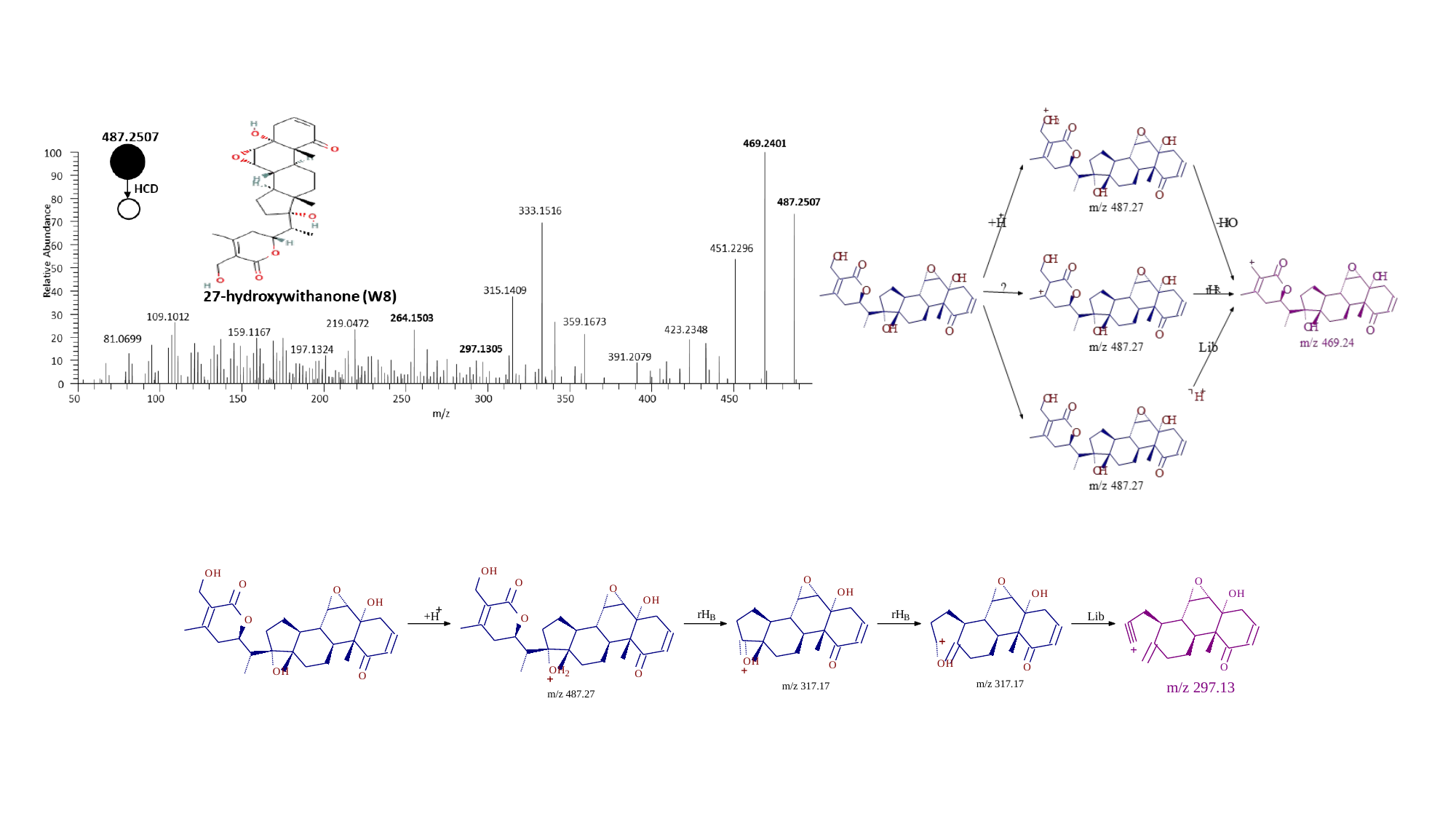

O
H
O
H
O
O
O
O
O
O
O
H
O
H
O
H
O
H
r
H
+H
O
B
O
O
H
O
H
O
O
O
H
O
H
O
2
O
m/z 317.17
m/z 317.17
m/z 487.27
O
O
H
r
H
Lib
B
O
m/z 297.13

## Slide 9
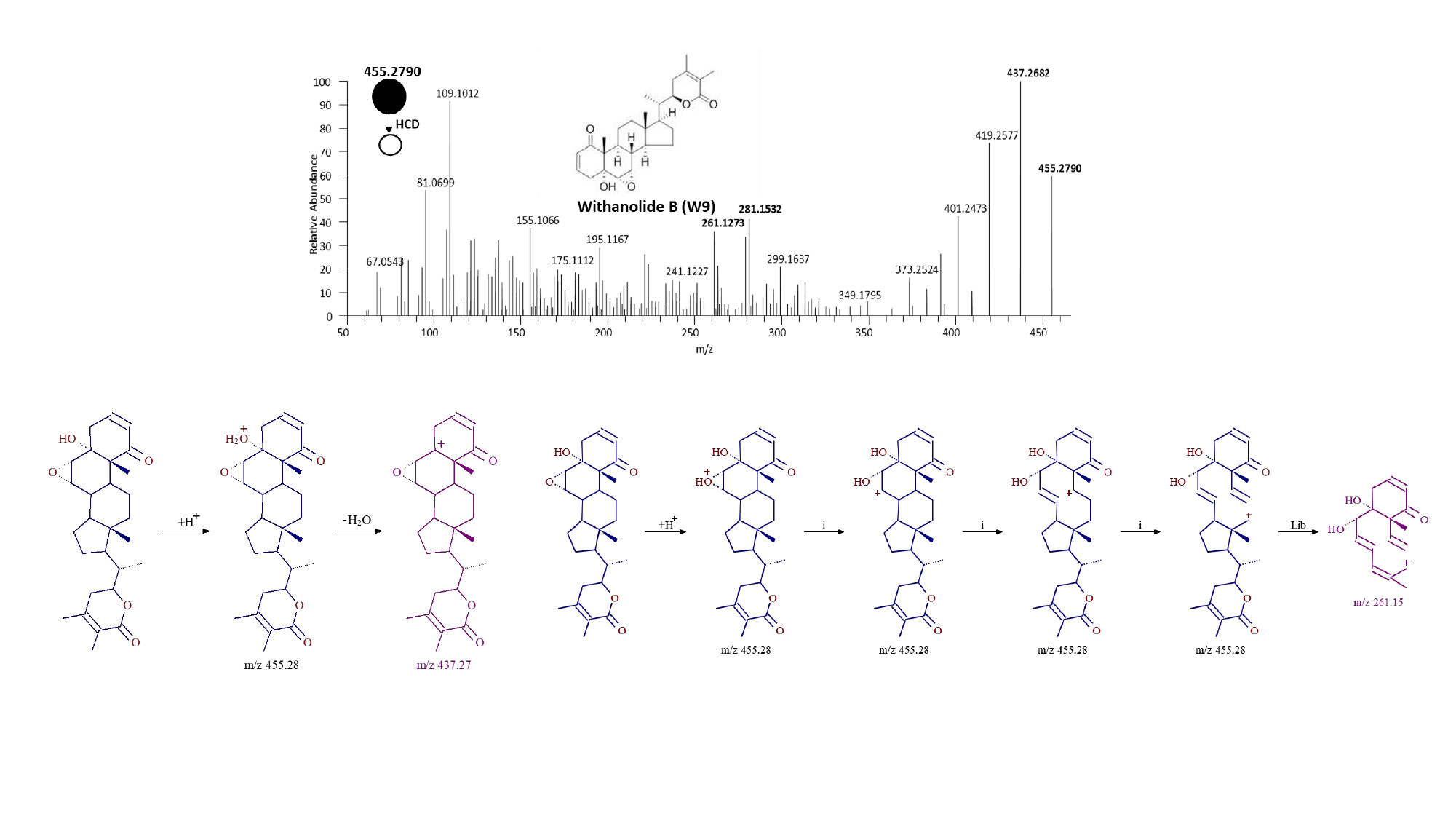

## Slide 10
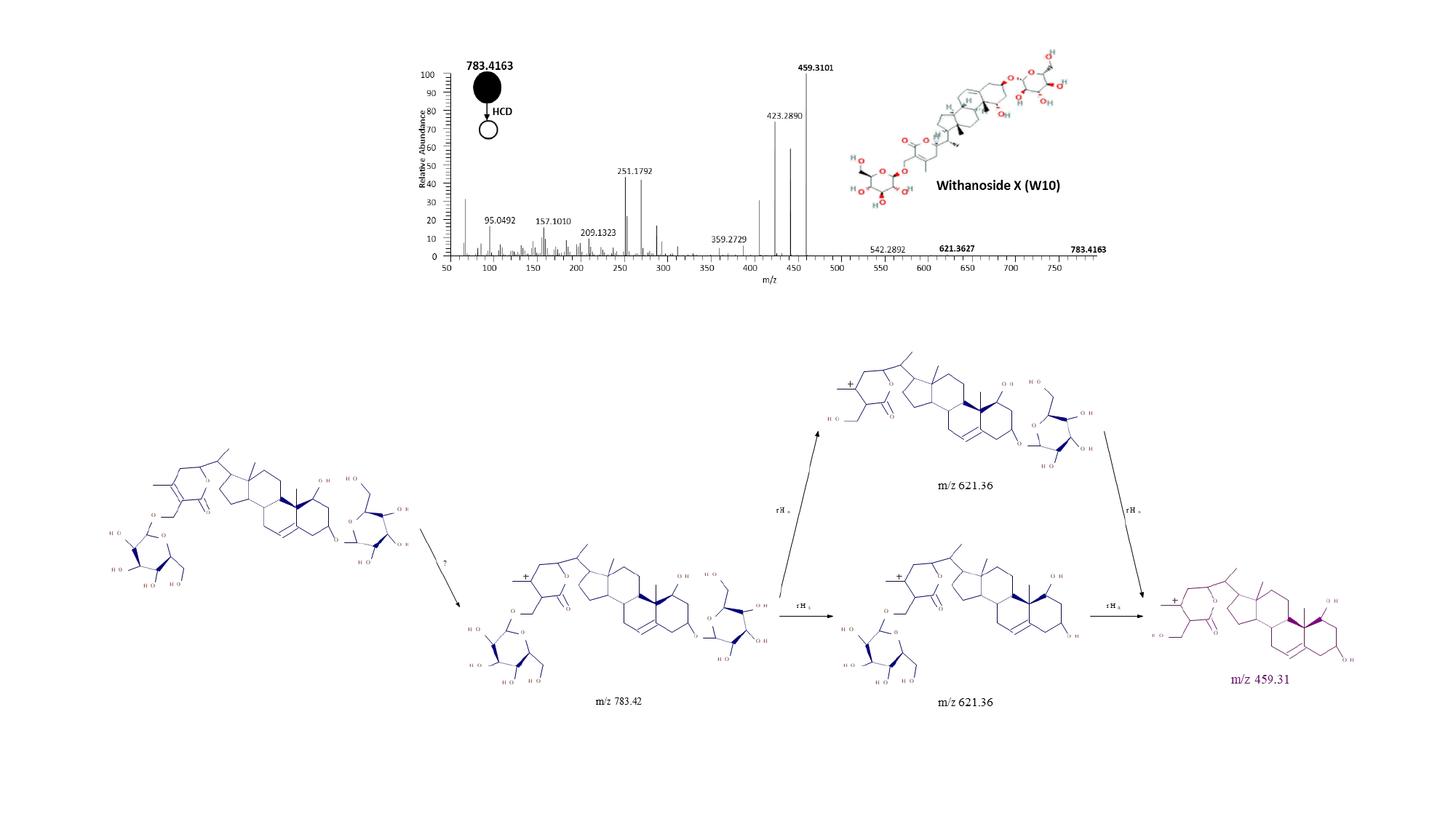

## Slide 11
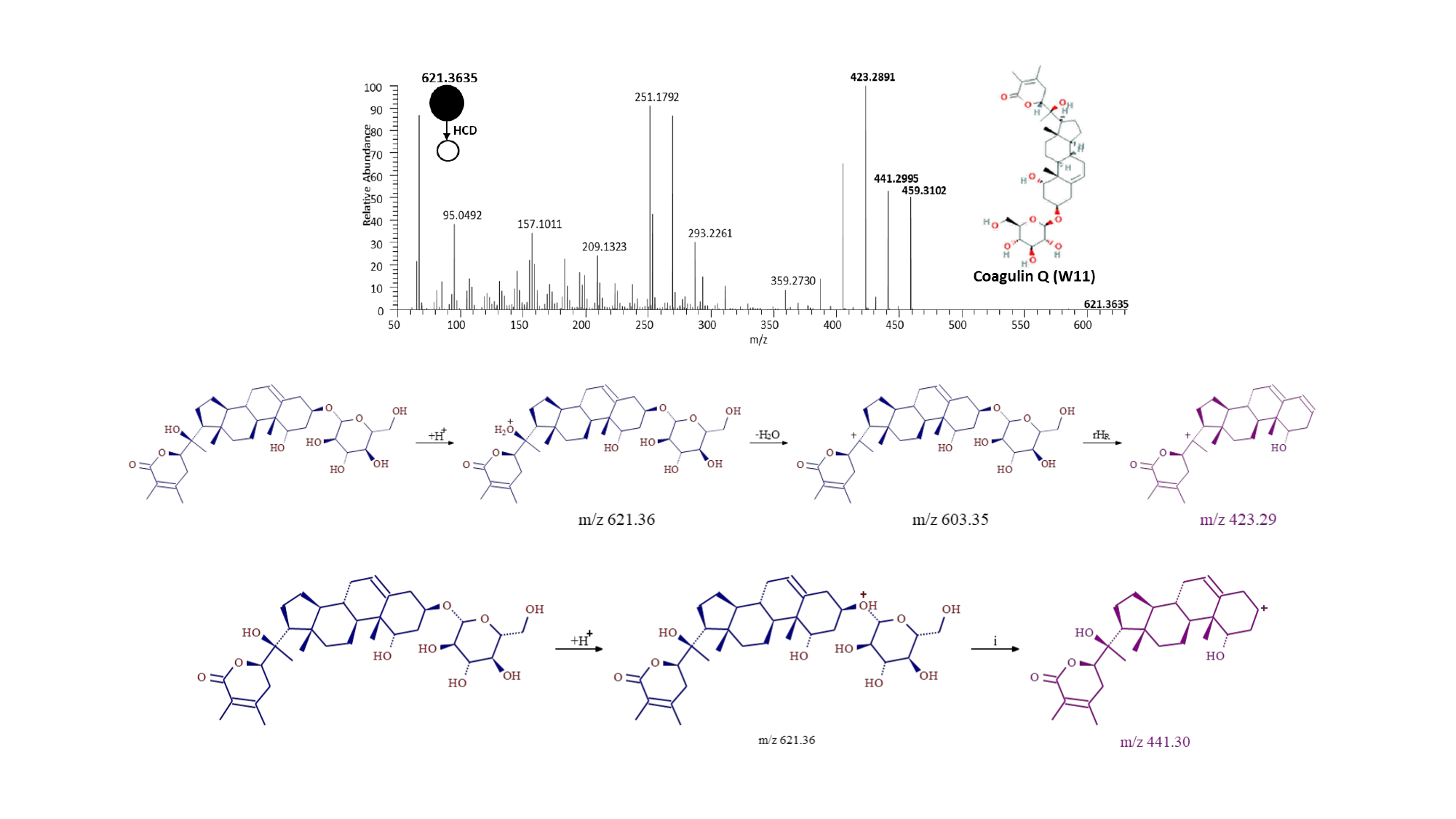

## Slide 12
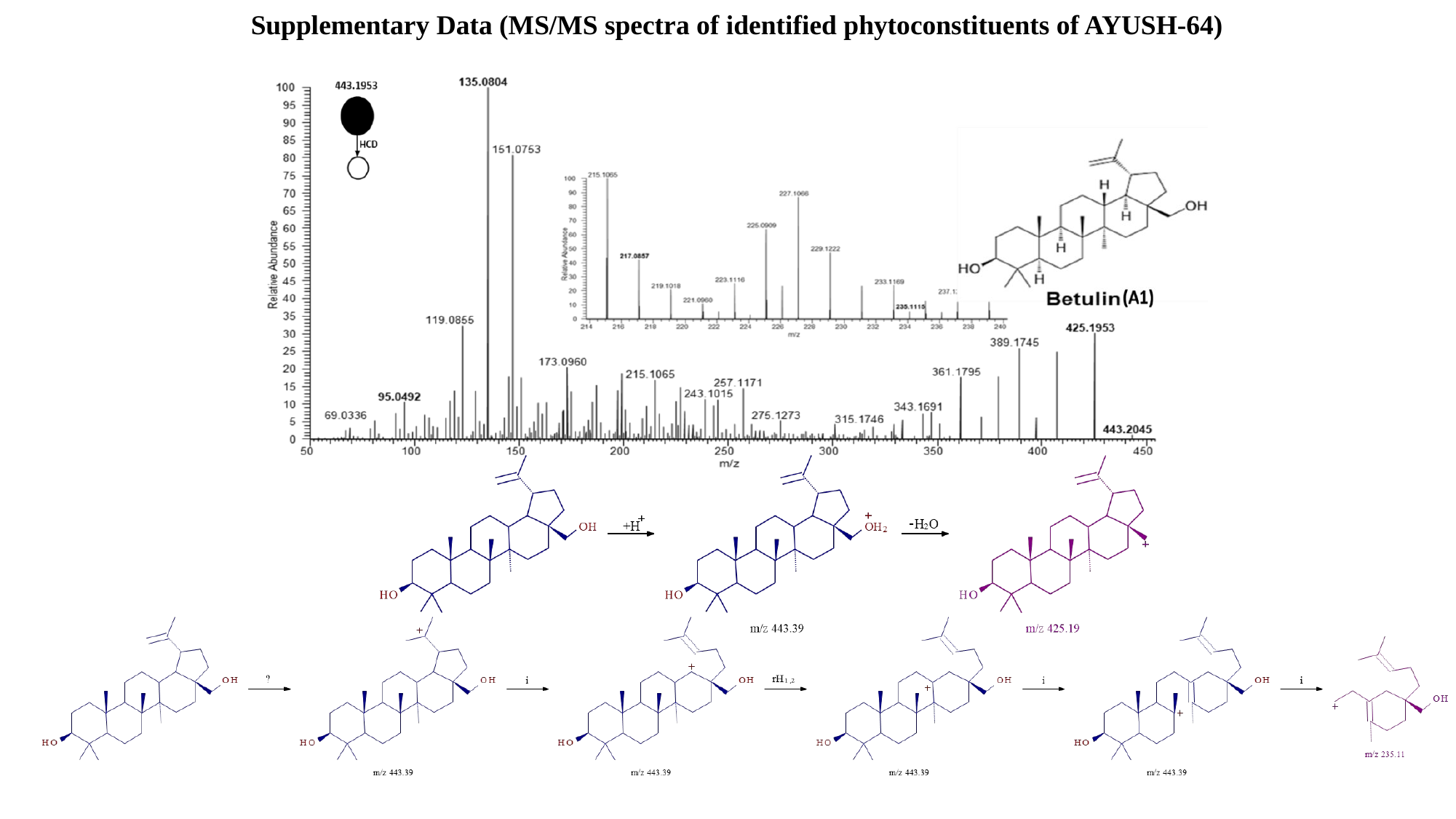

Supplementary Data (MS/MS spectra of identified phytoconstituents of AYUSH-64)

## Slide 13
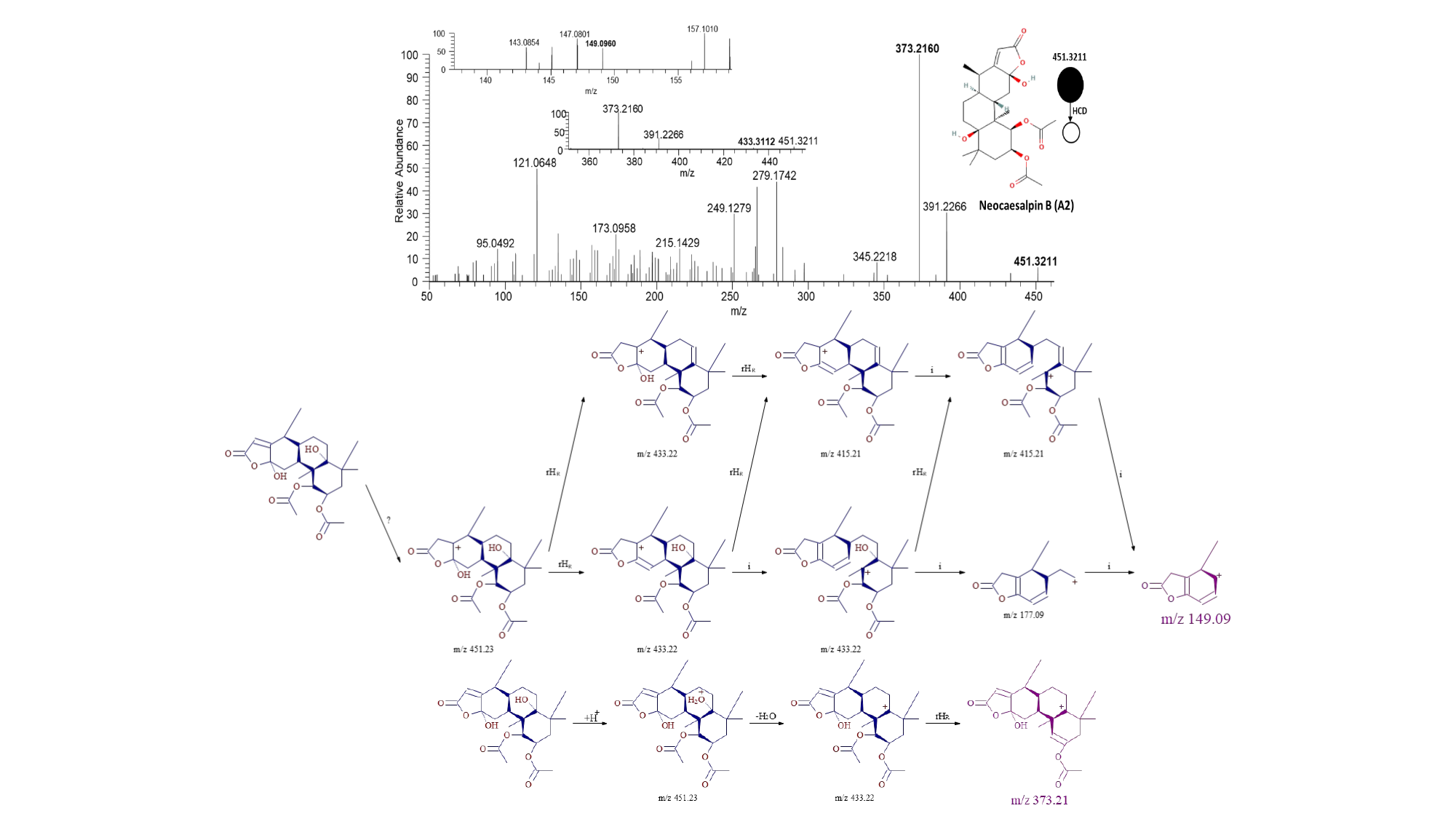

## Slide 14
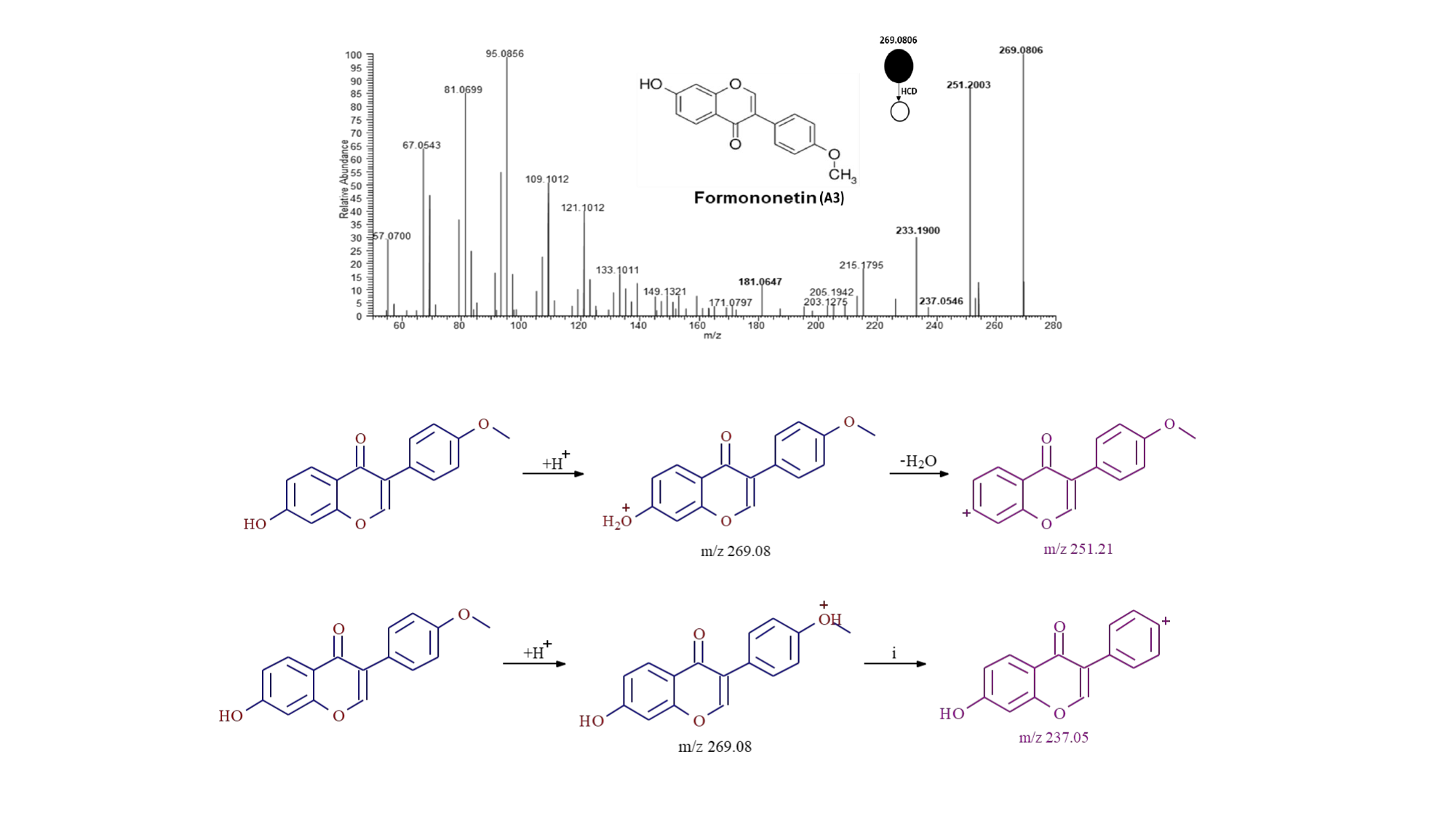

## Slide 15
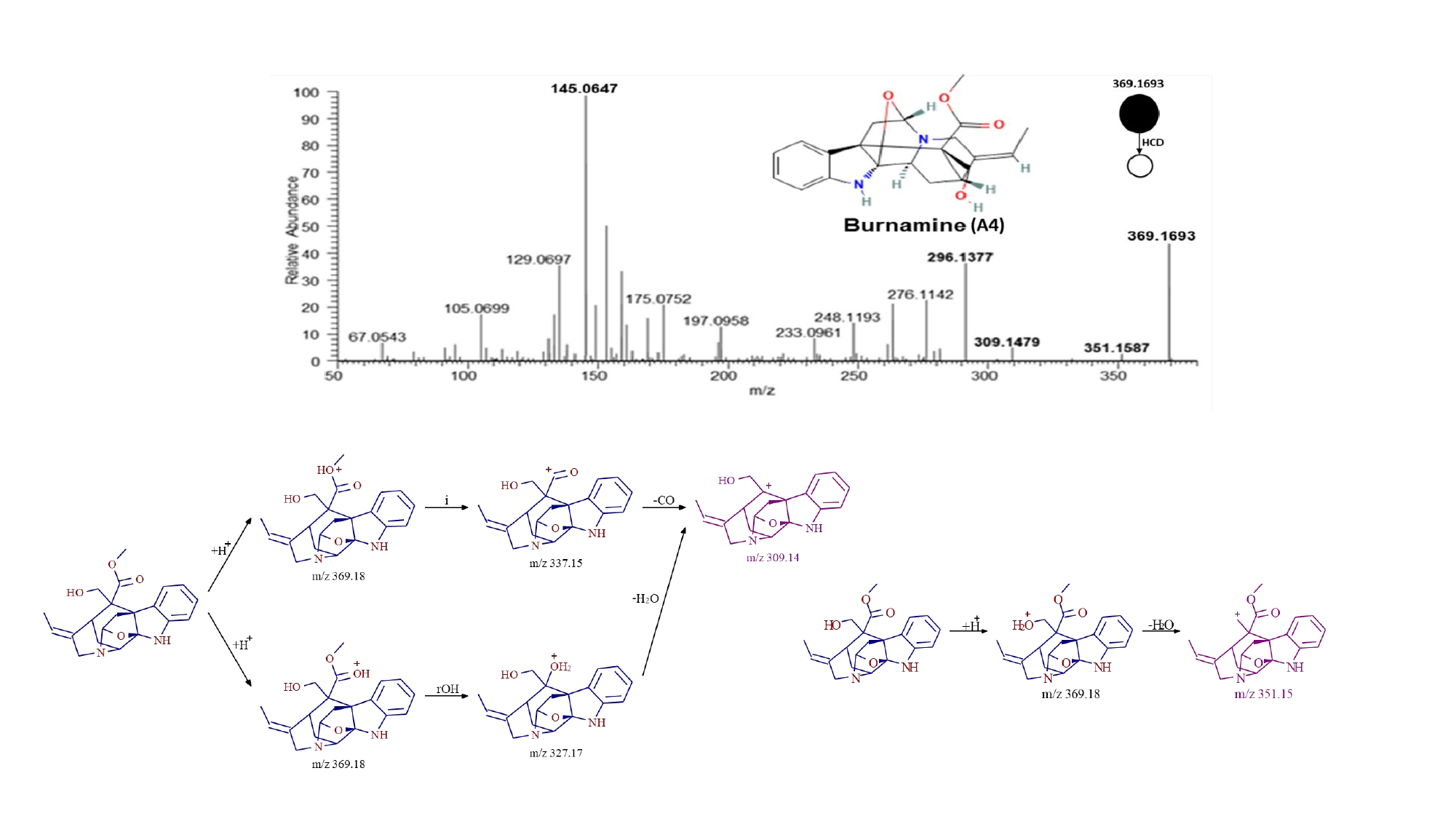

## Slide 16
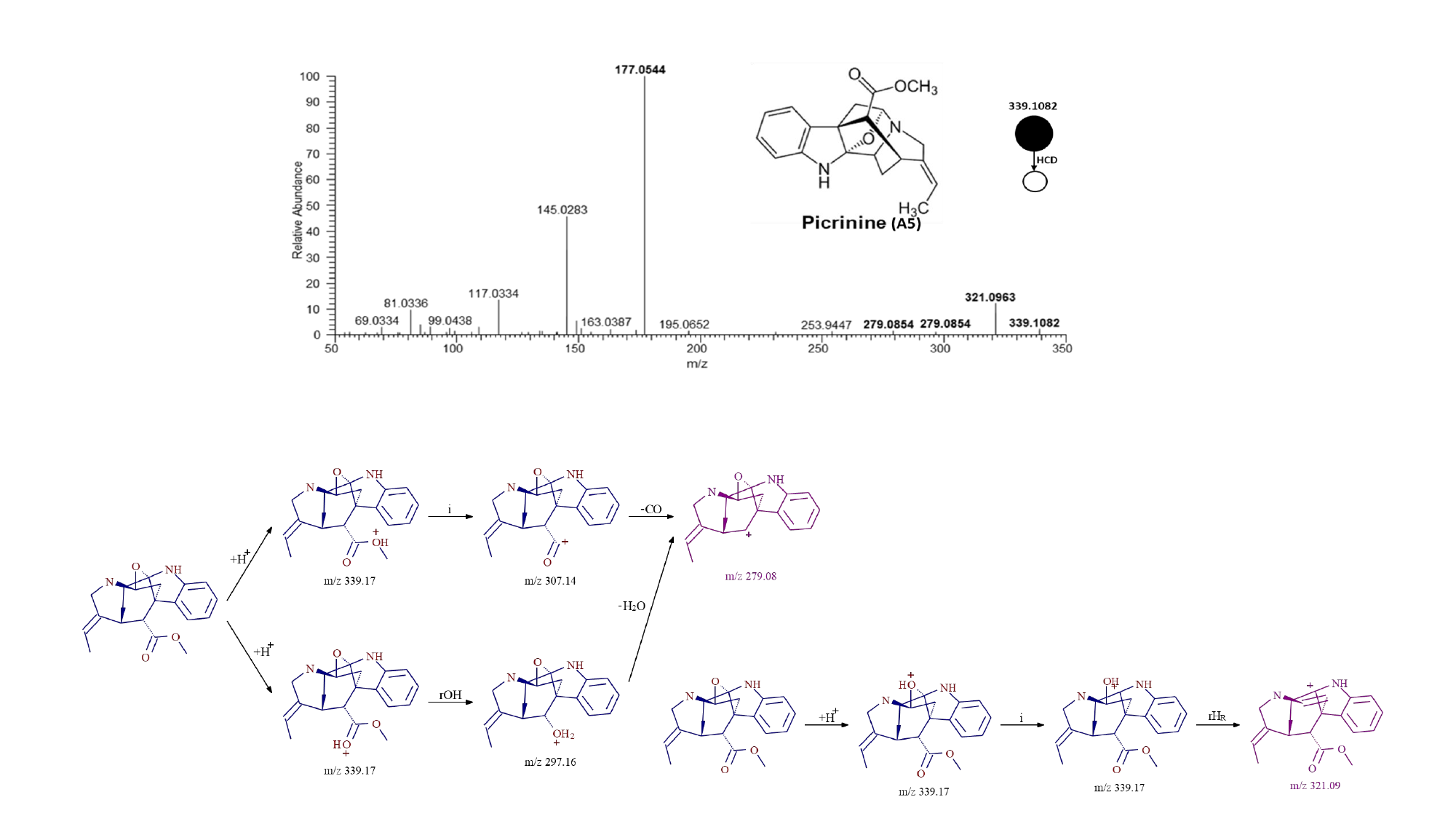

## Slide 17
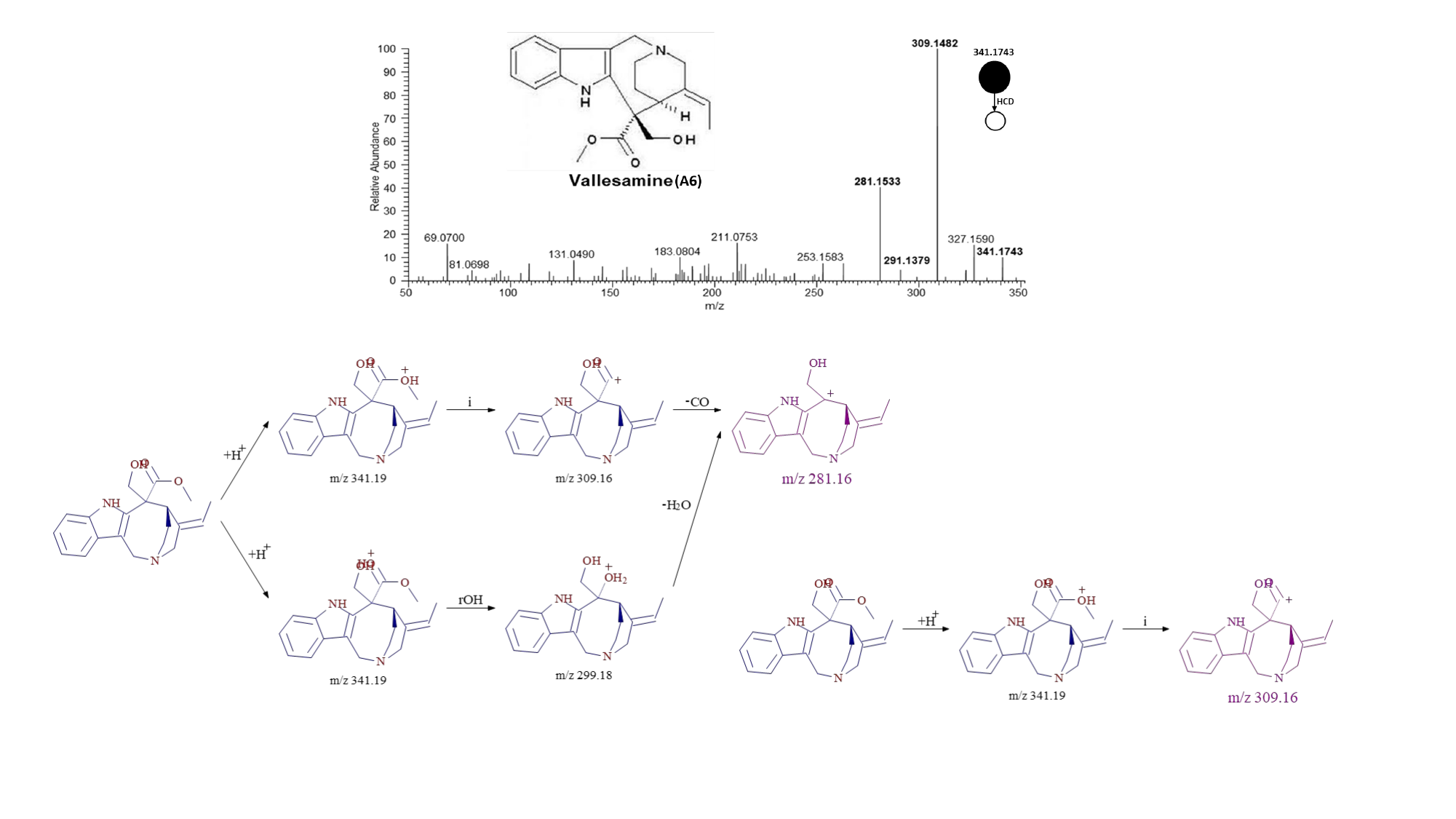

## Slide 18
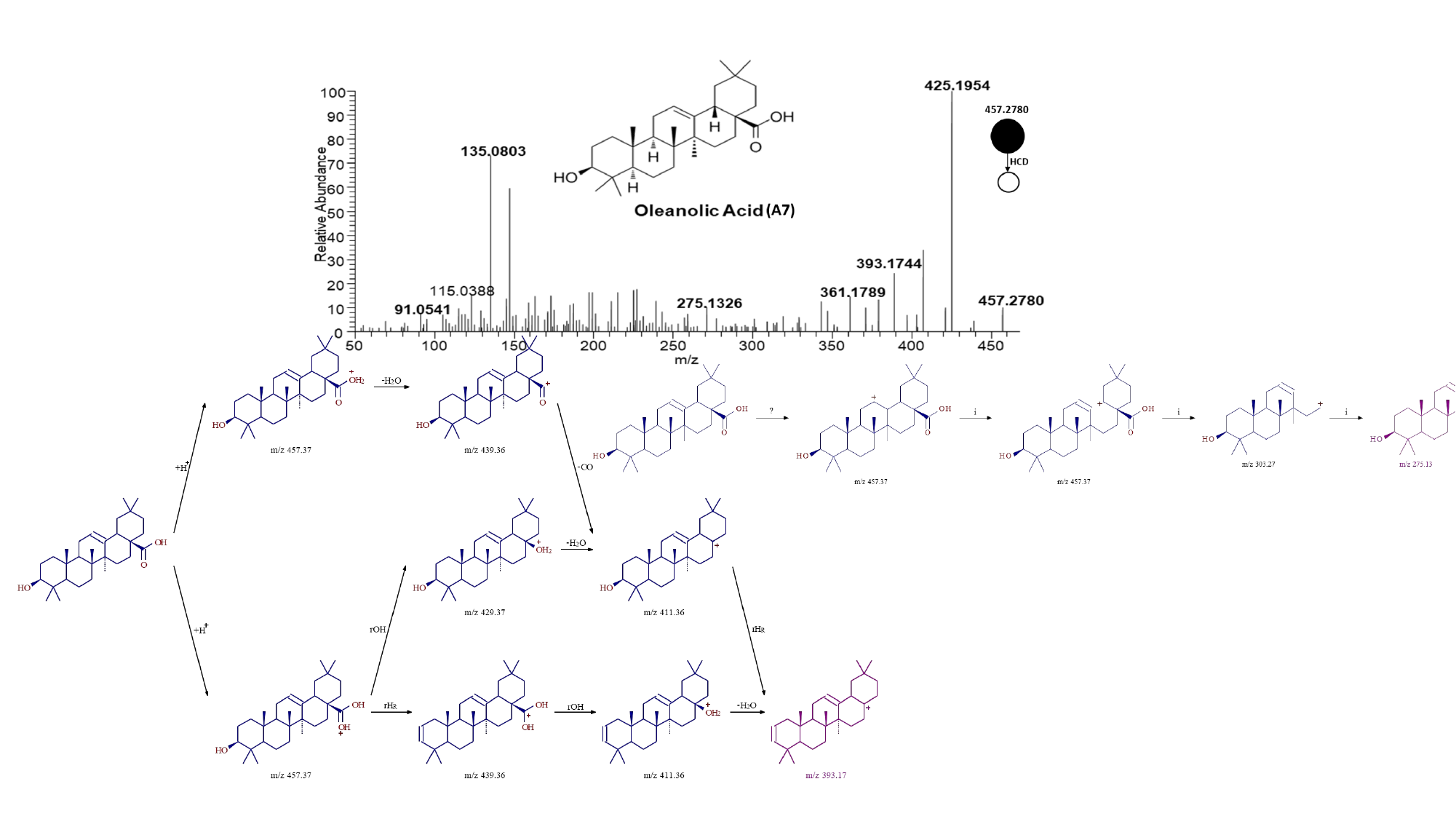

## Slide 19
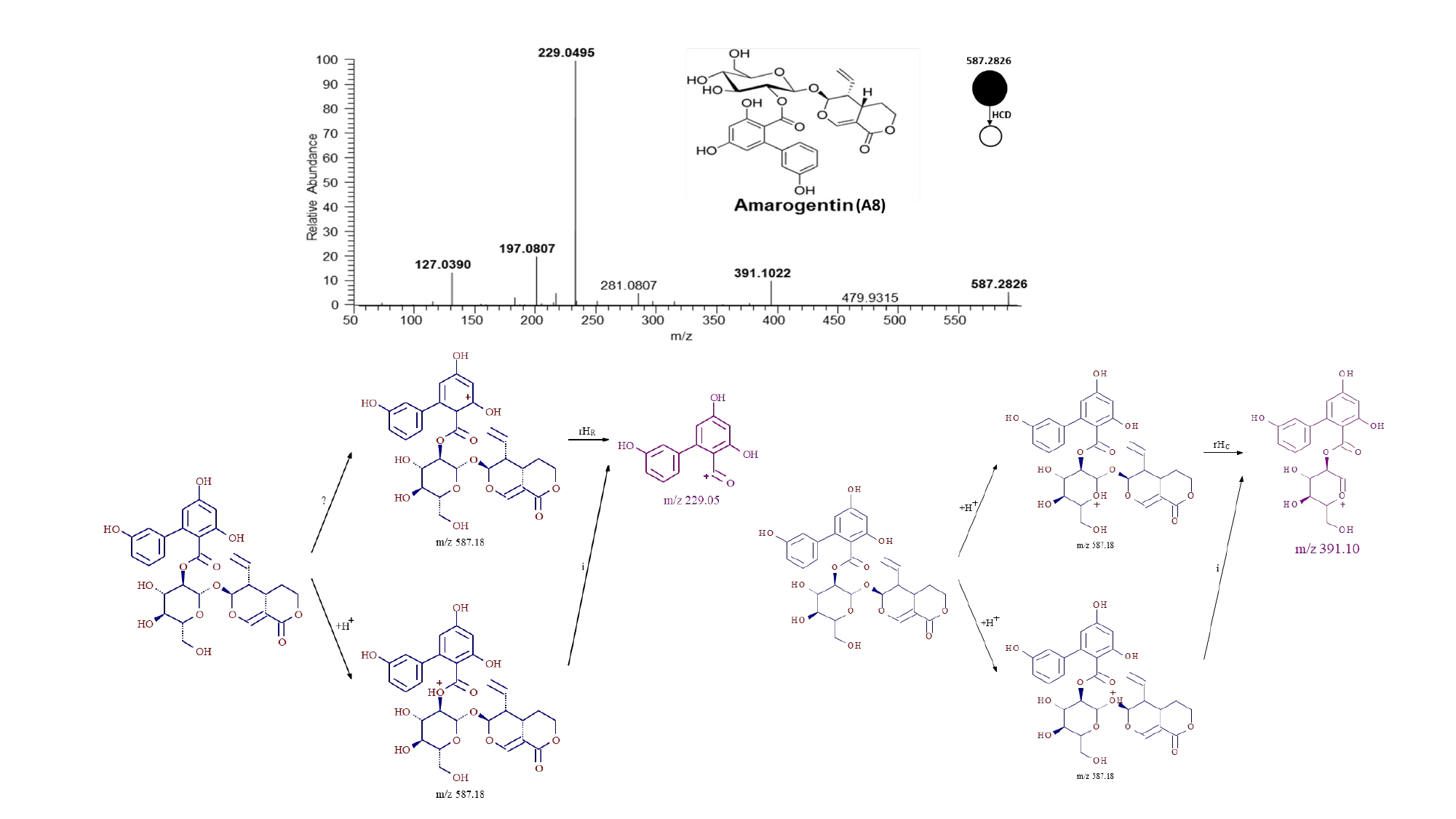

## Slide 20
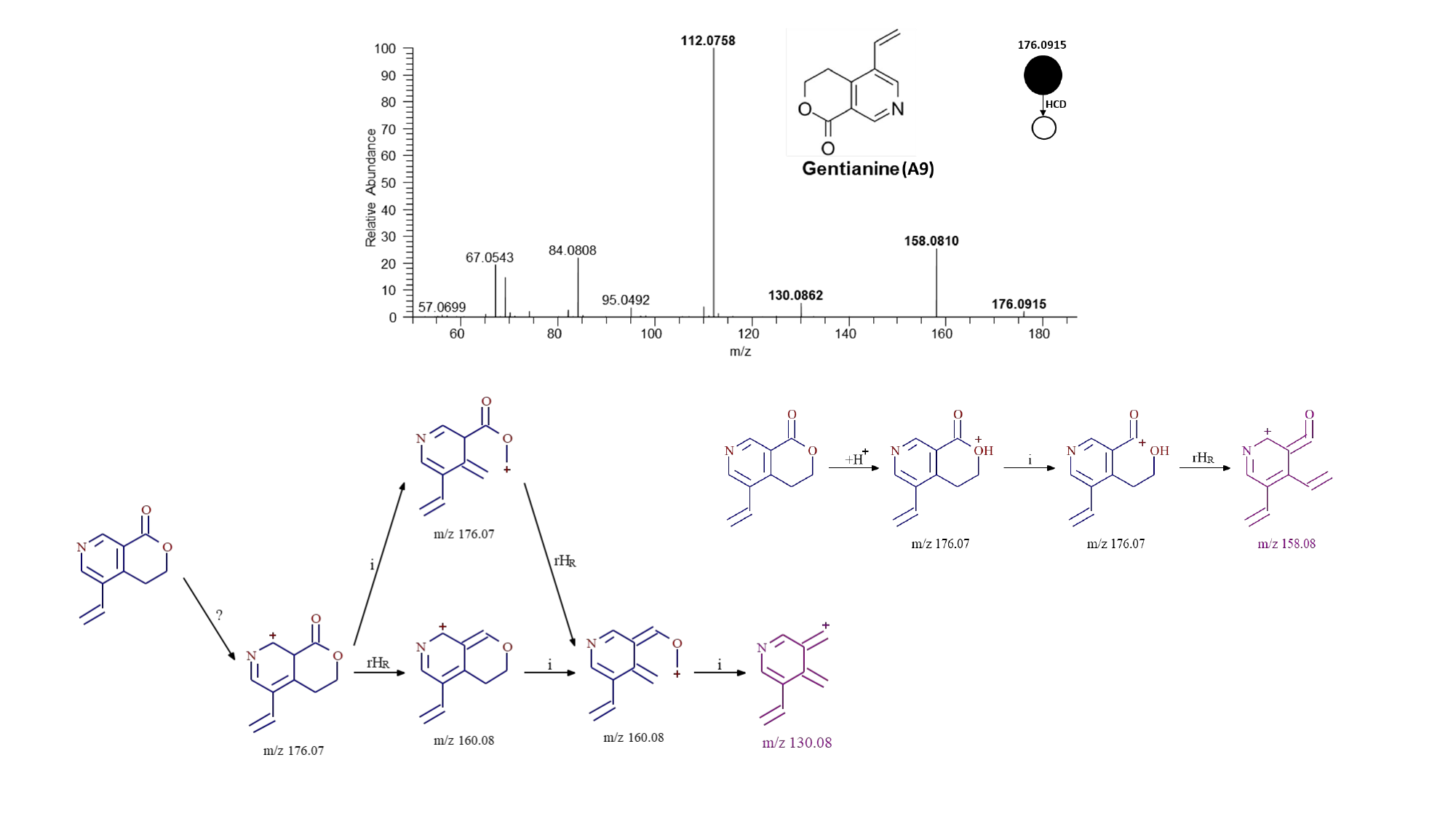

## Slide 21
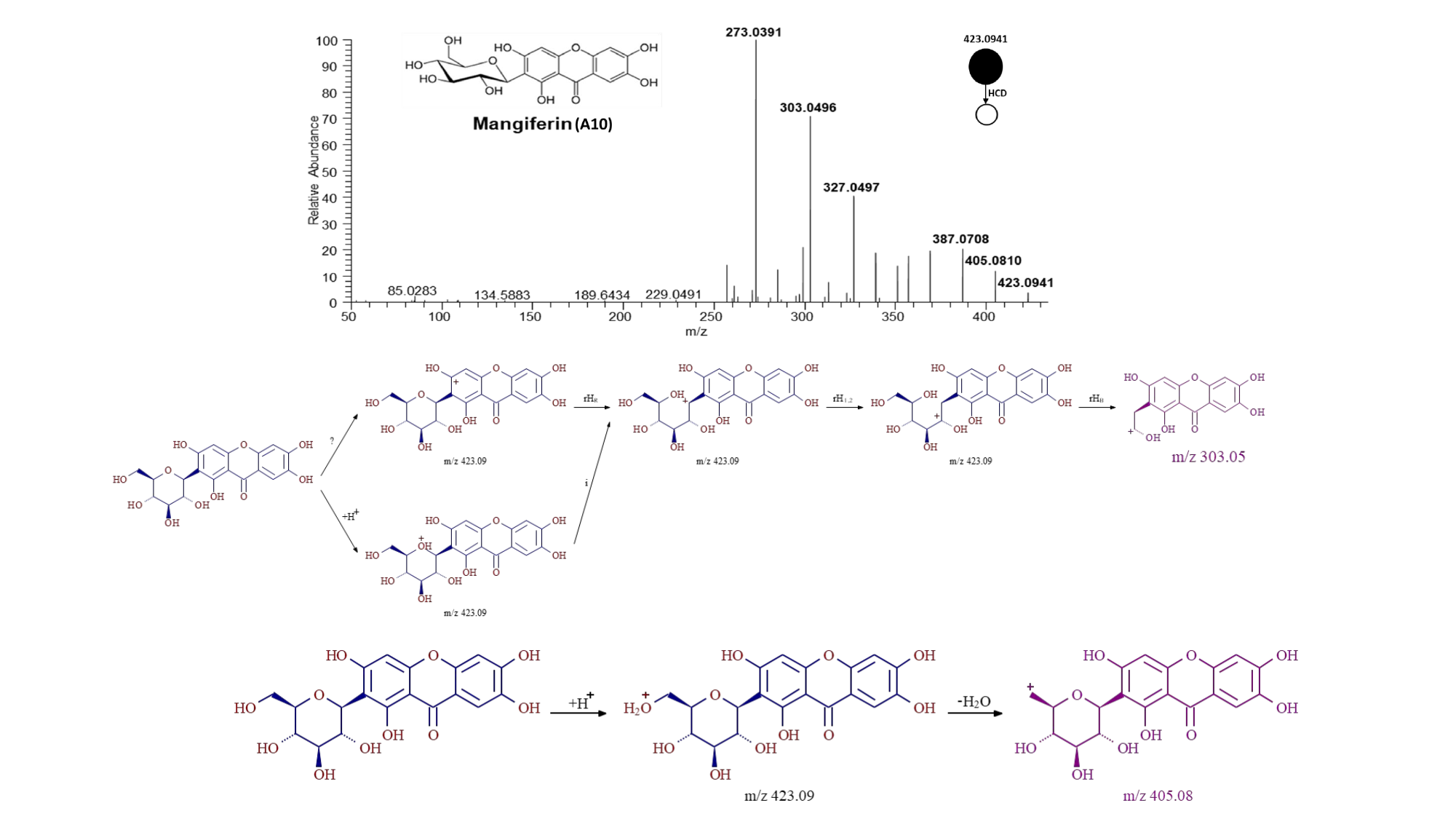

## Slide 22
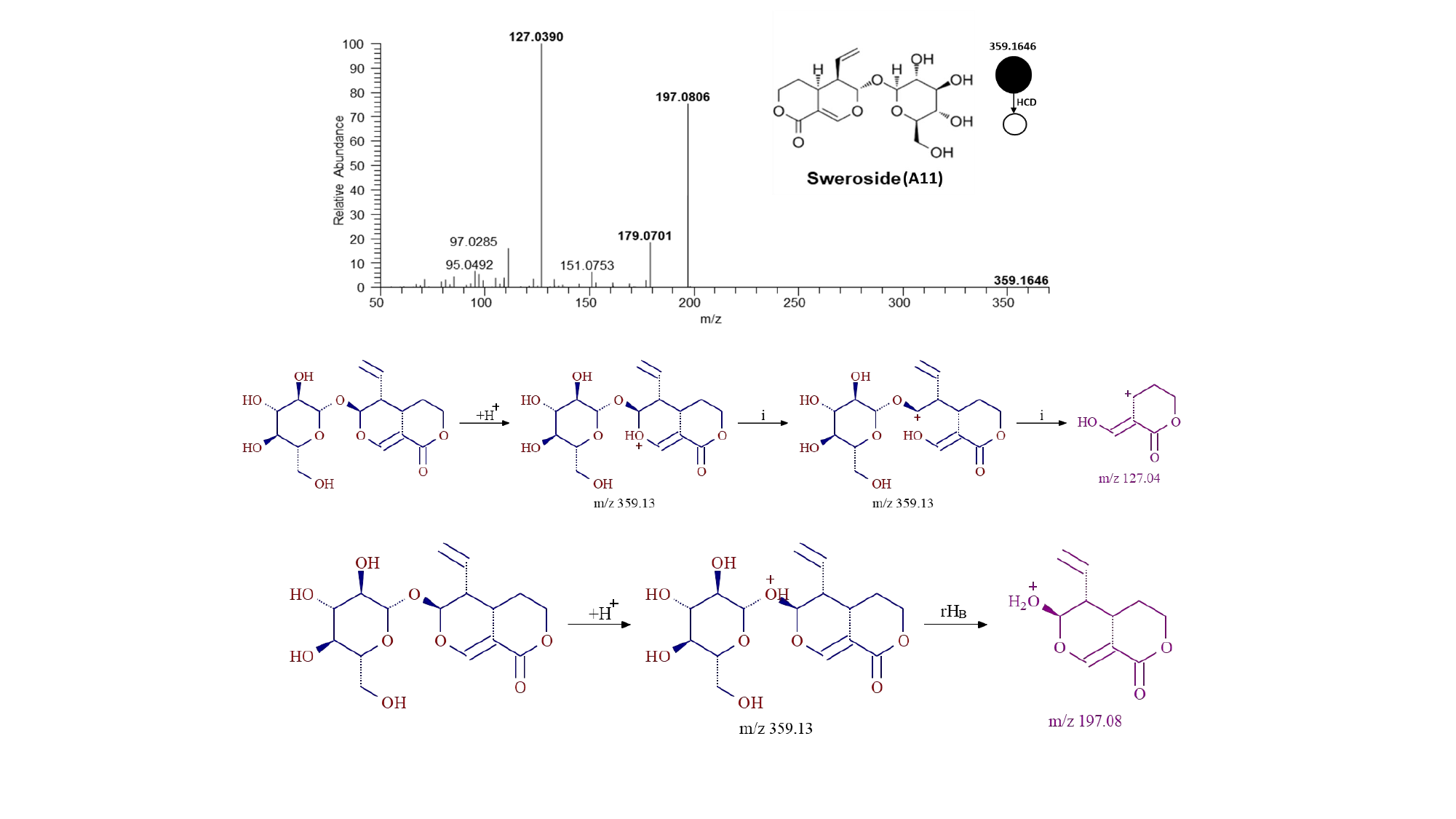

## Slide 23
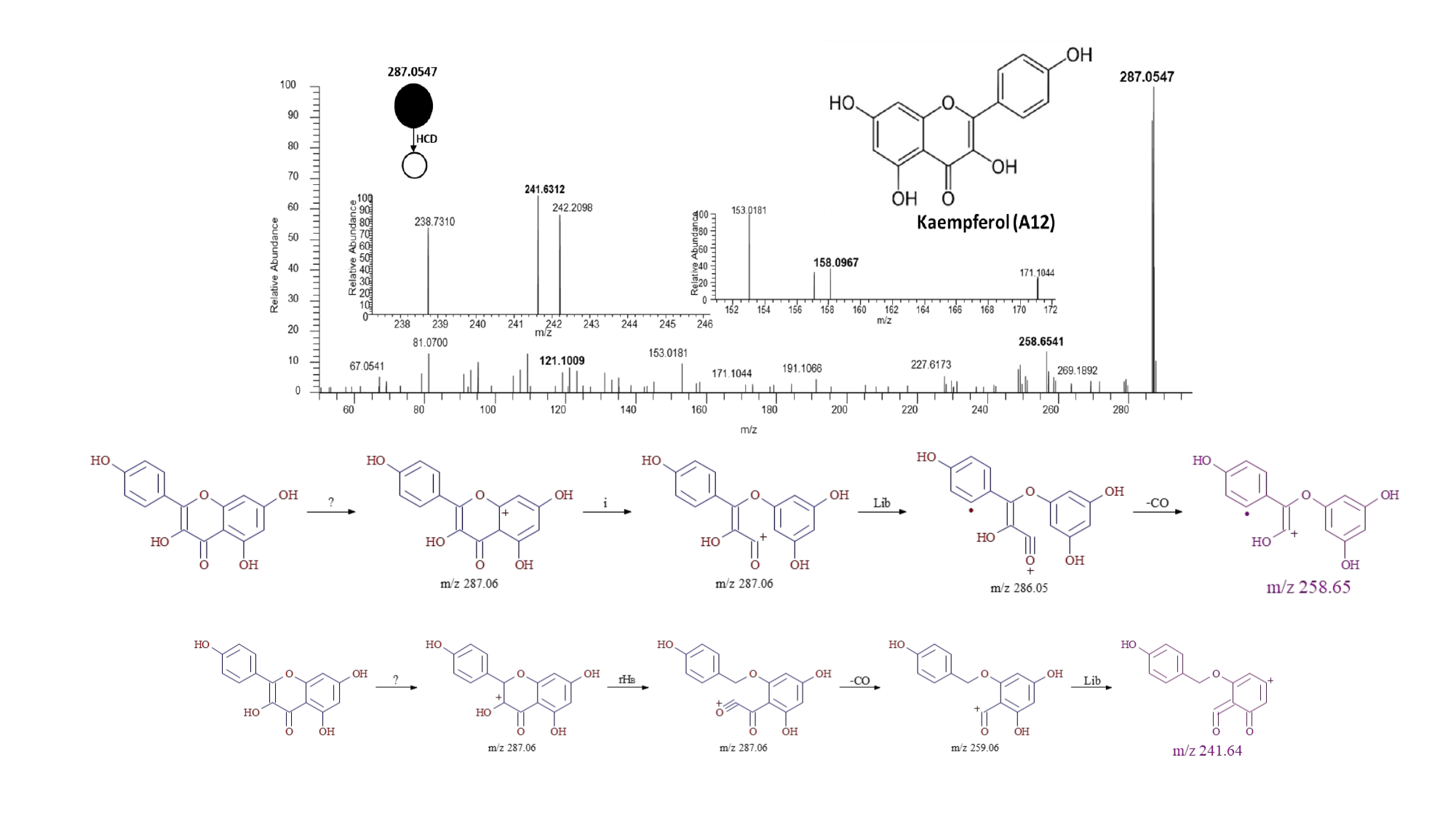

## Slide 24
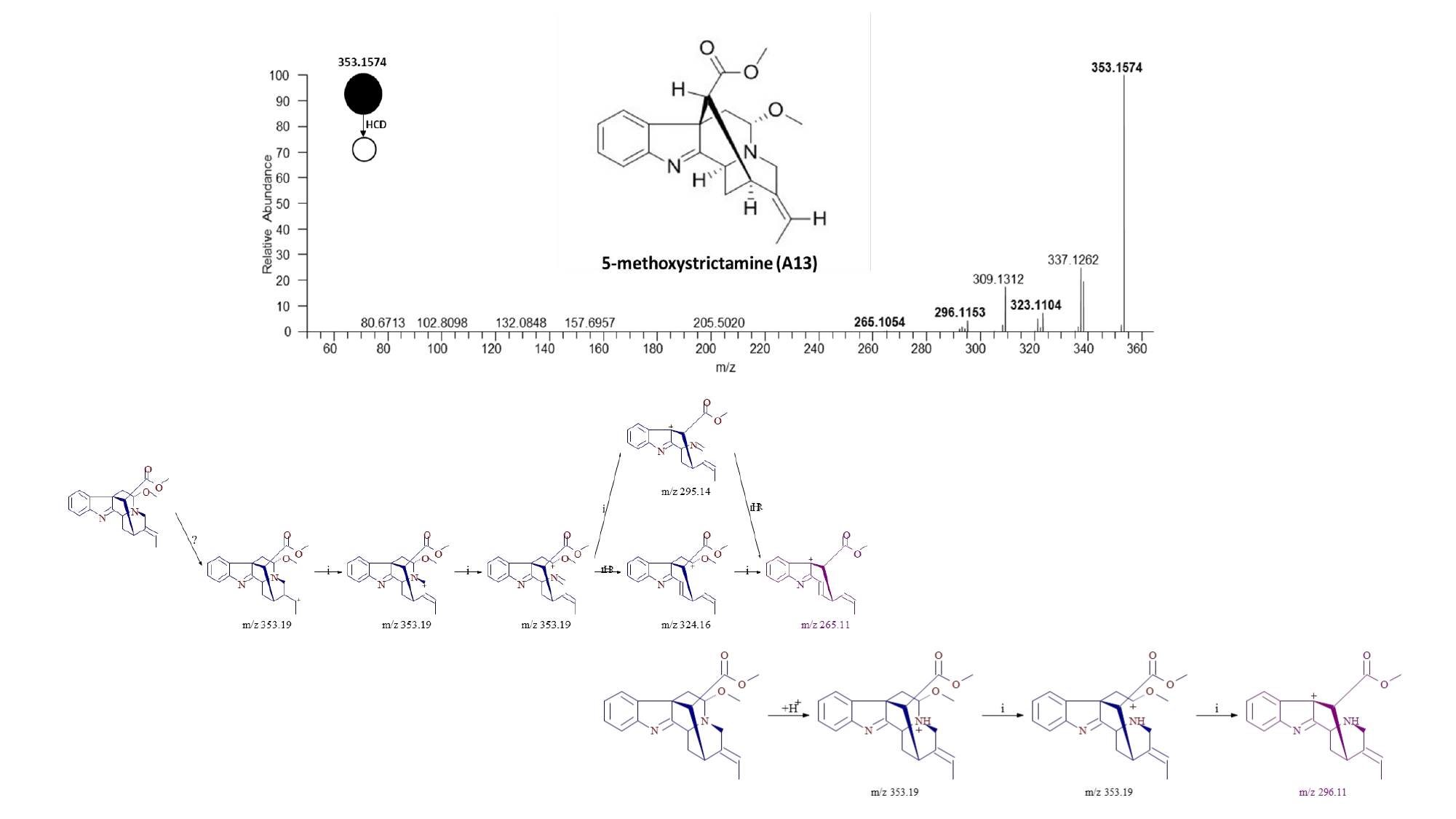

## Slide 25
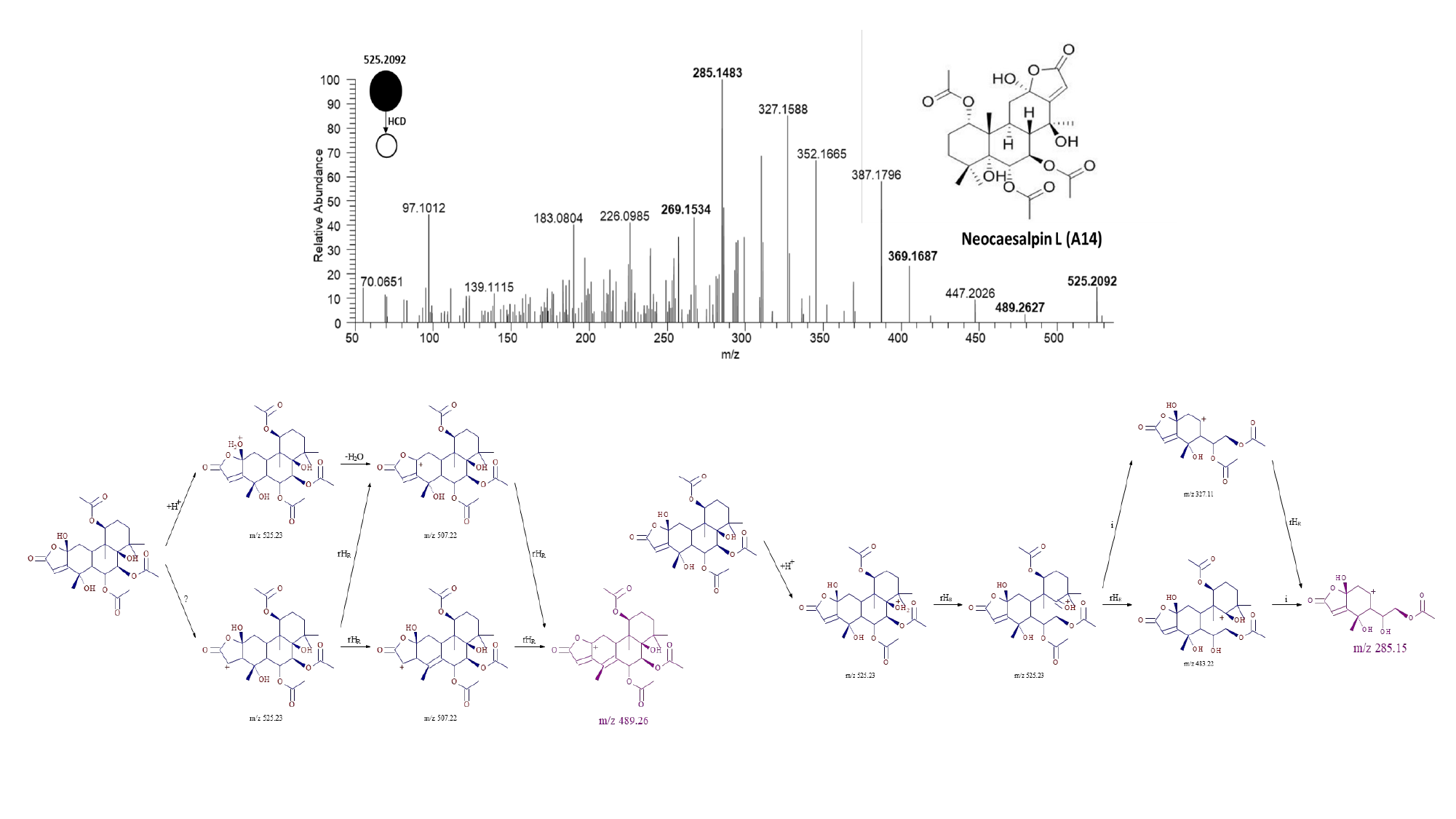

## Slide 26
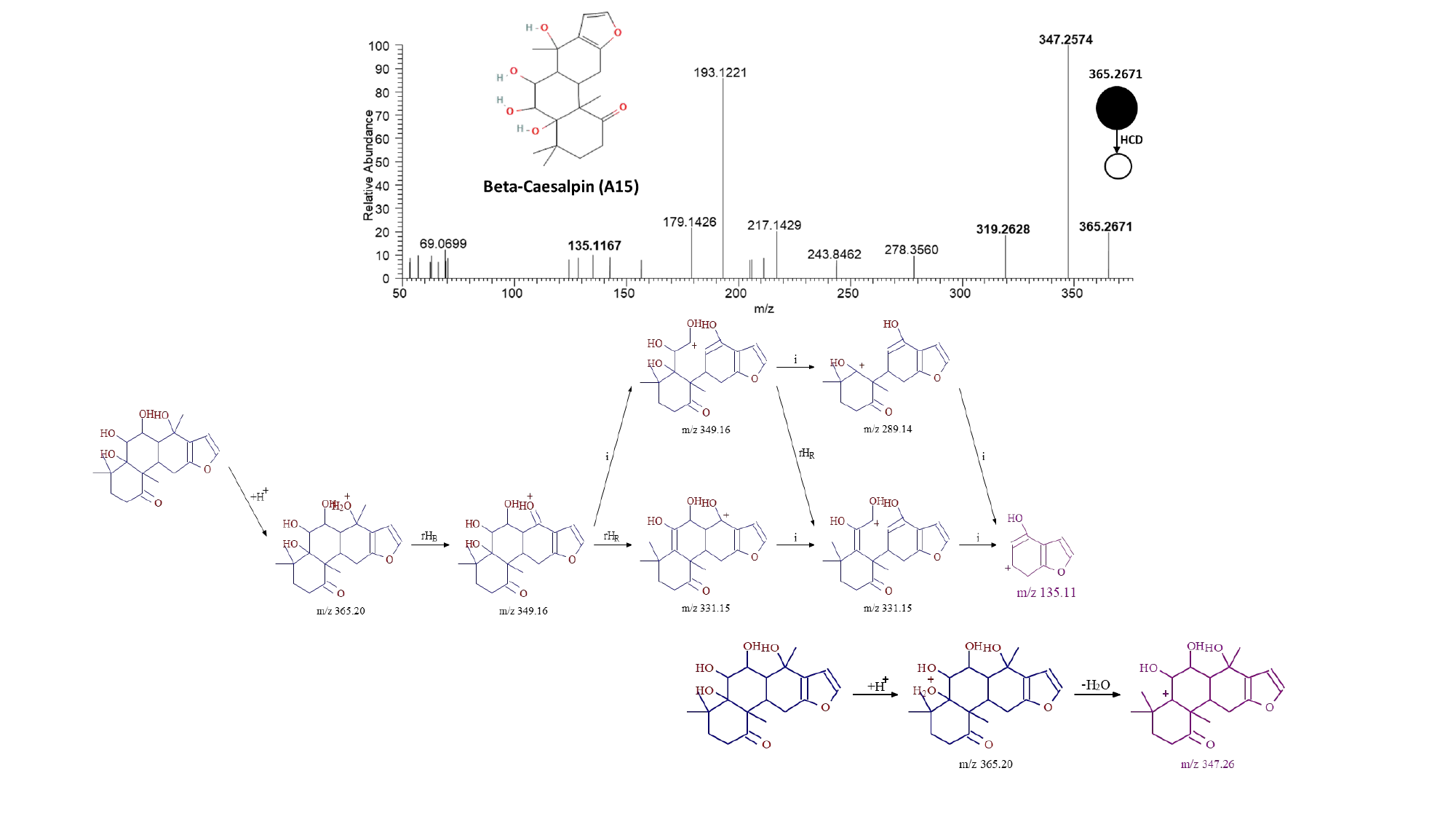

## Slide 27
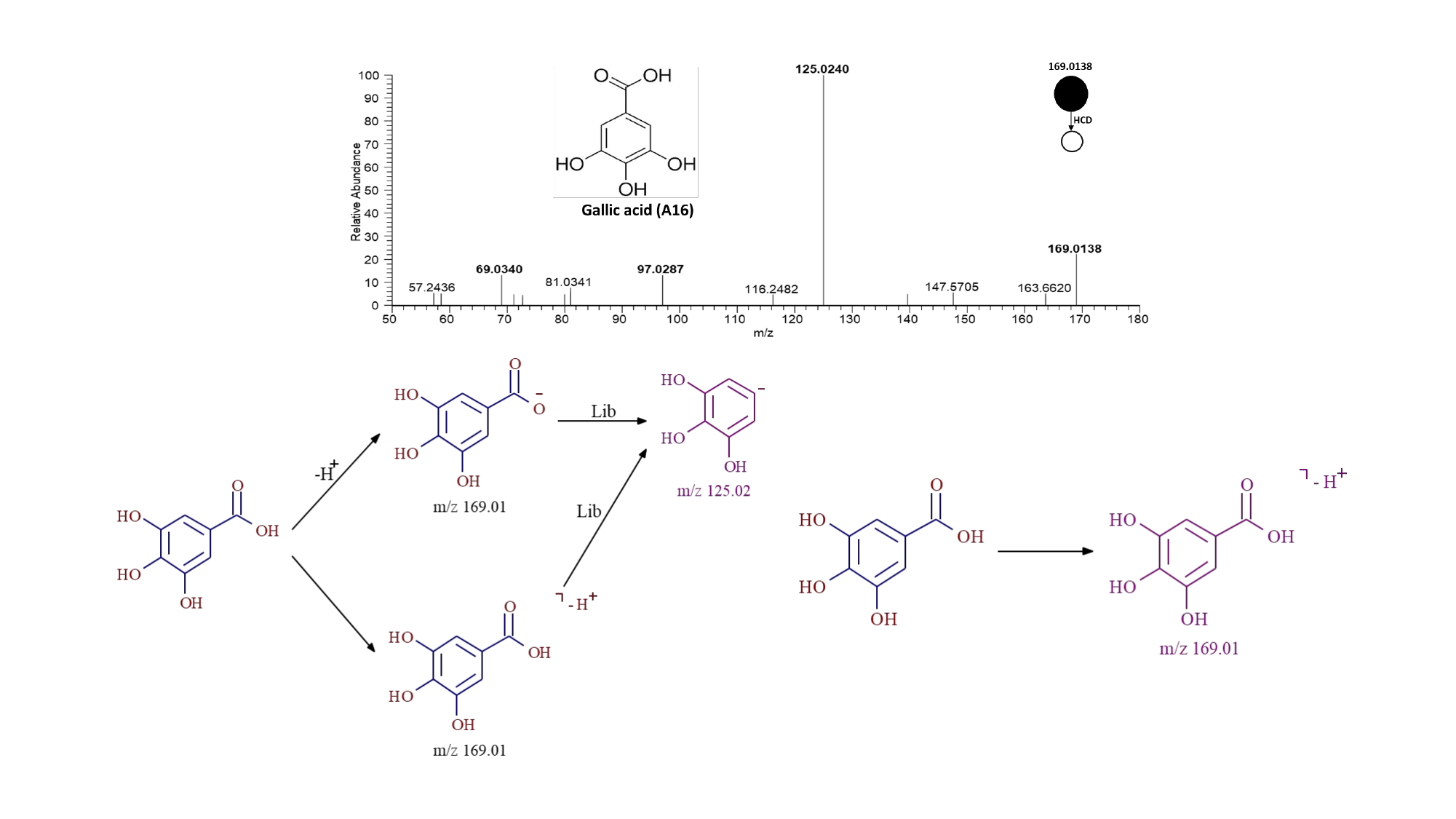

## Slide 28
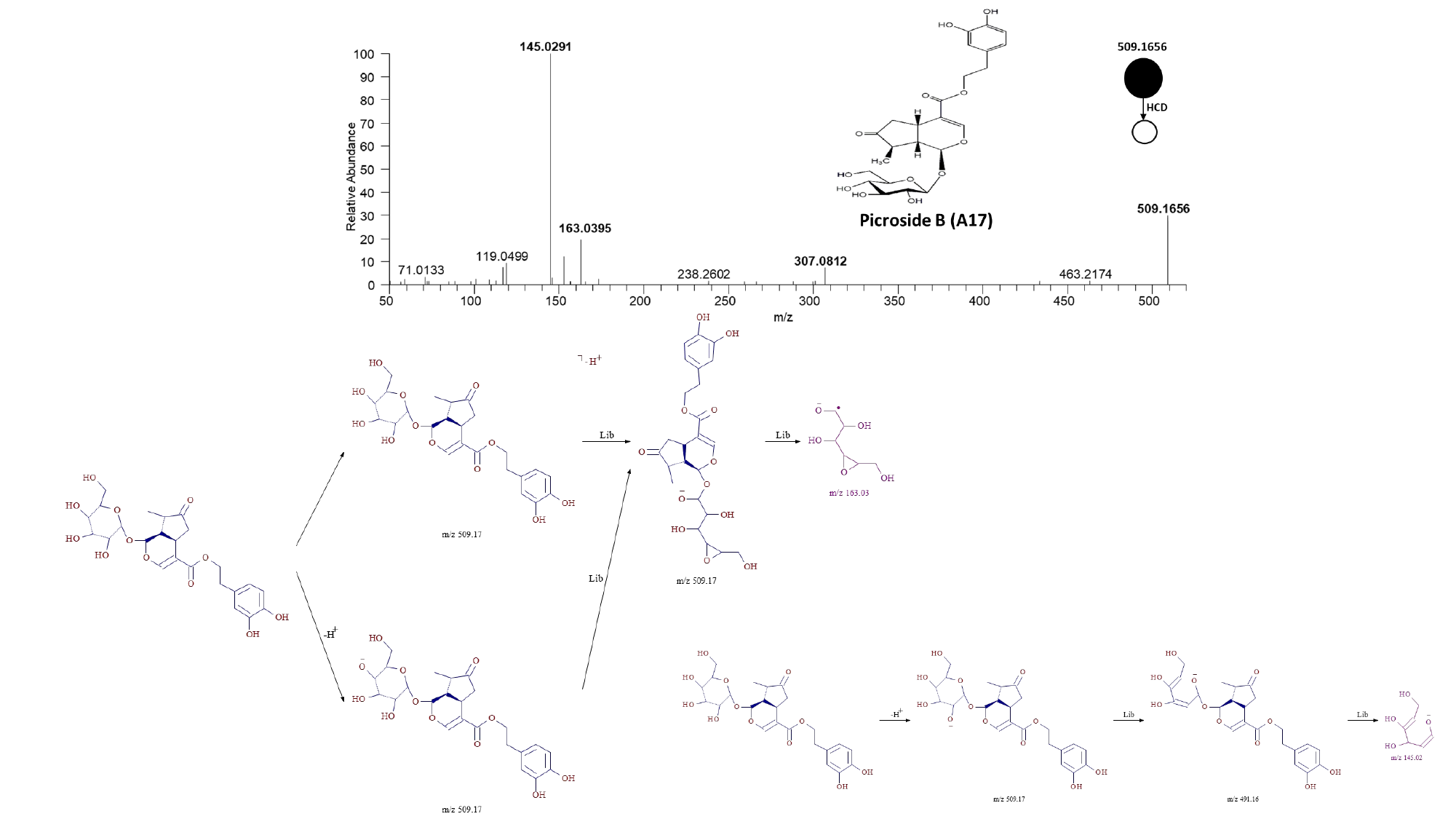

## Slide 29
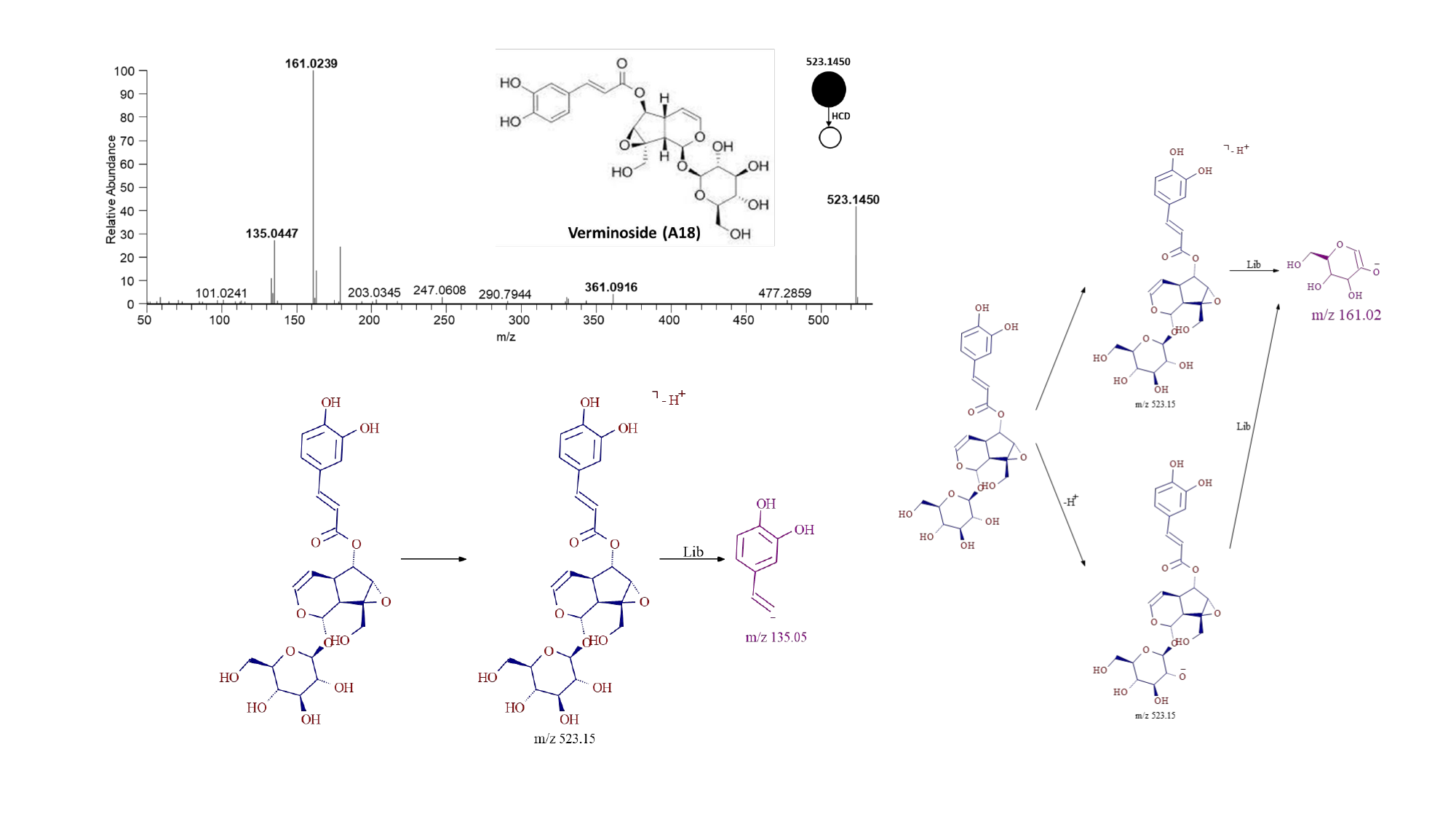

## Slide 30
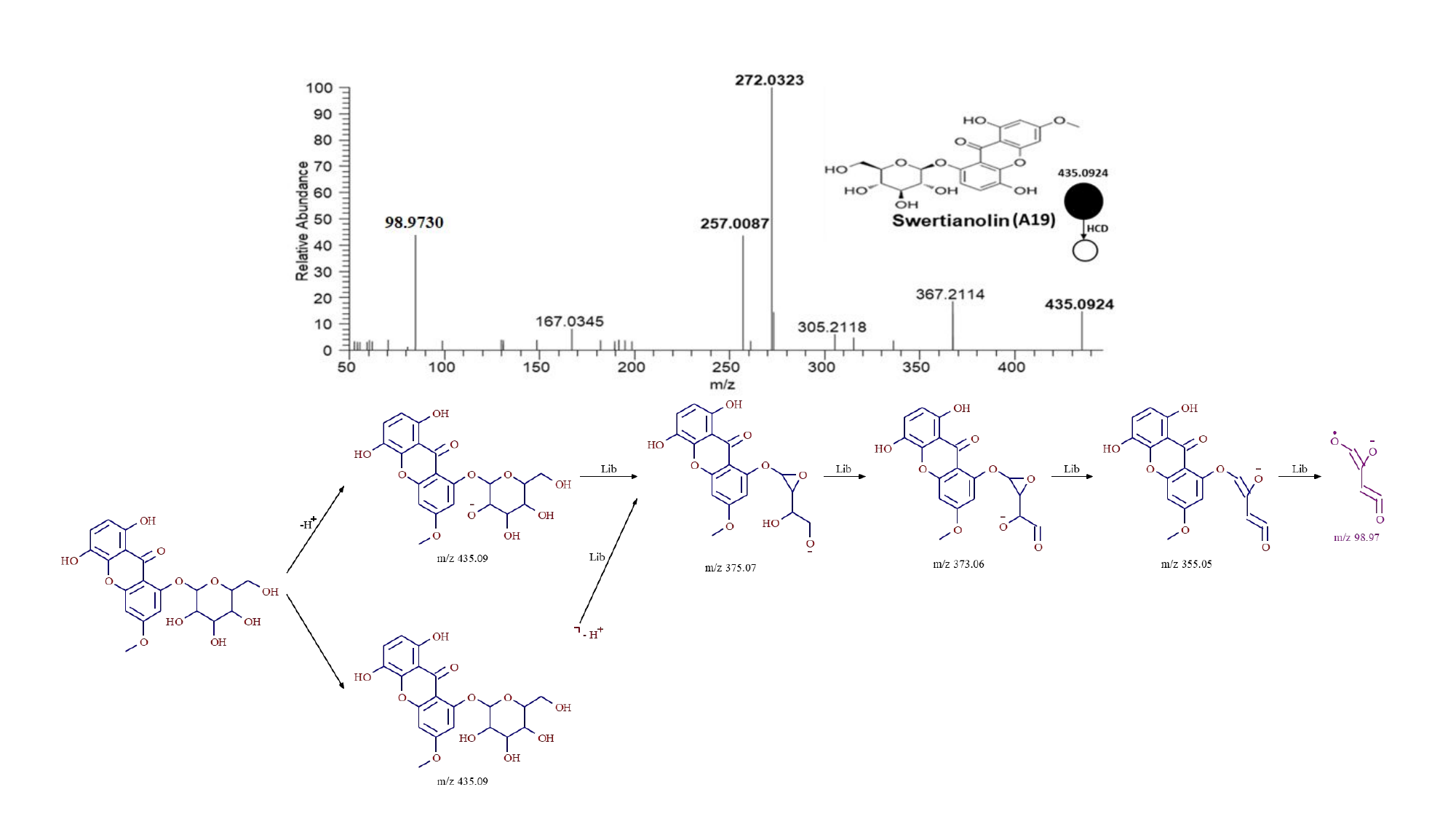

## Slide 31
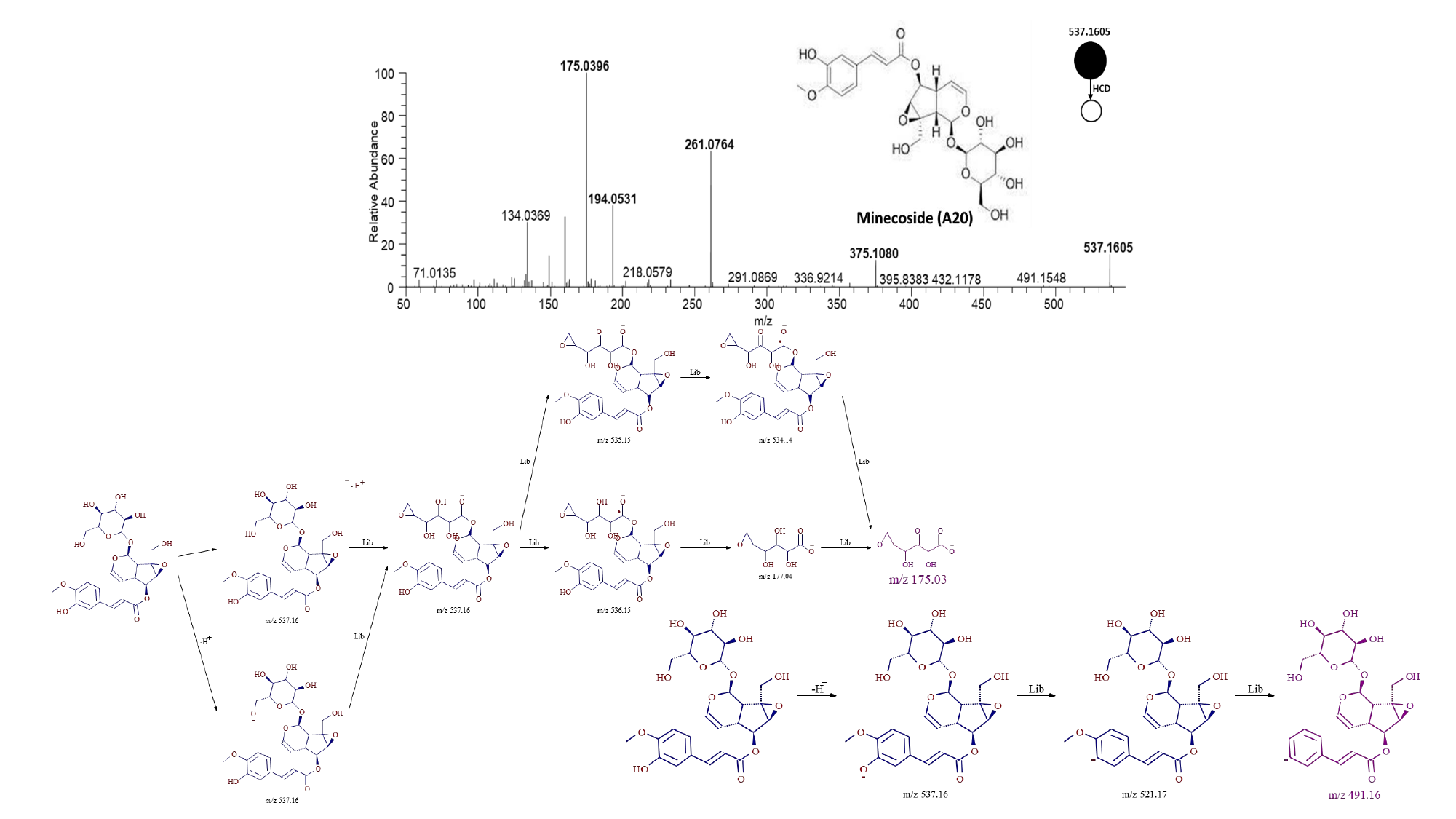

## Slide 32
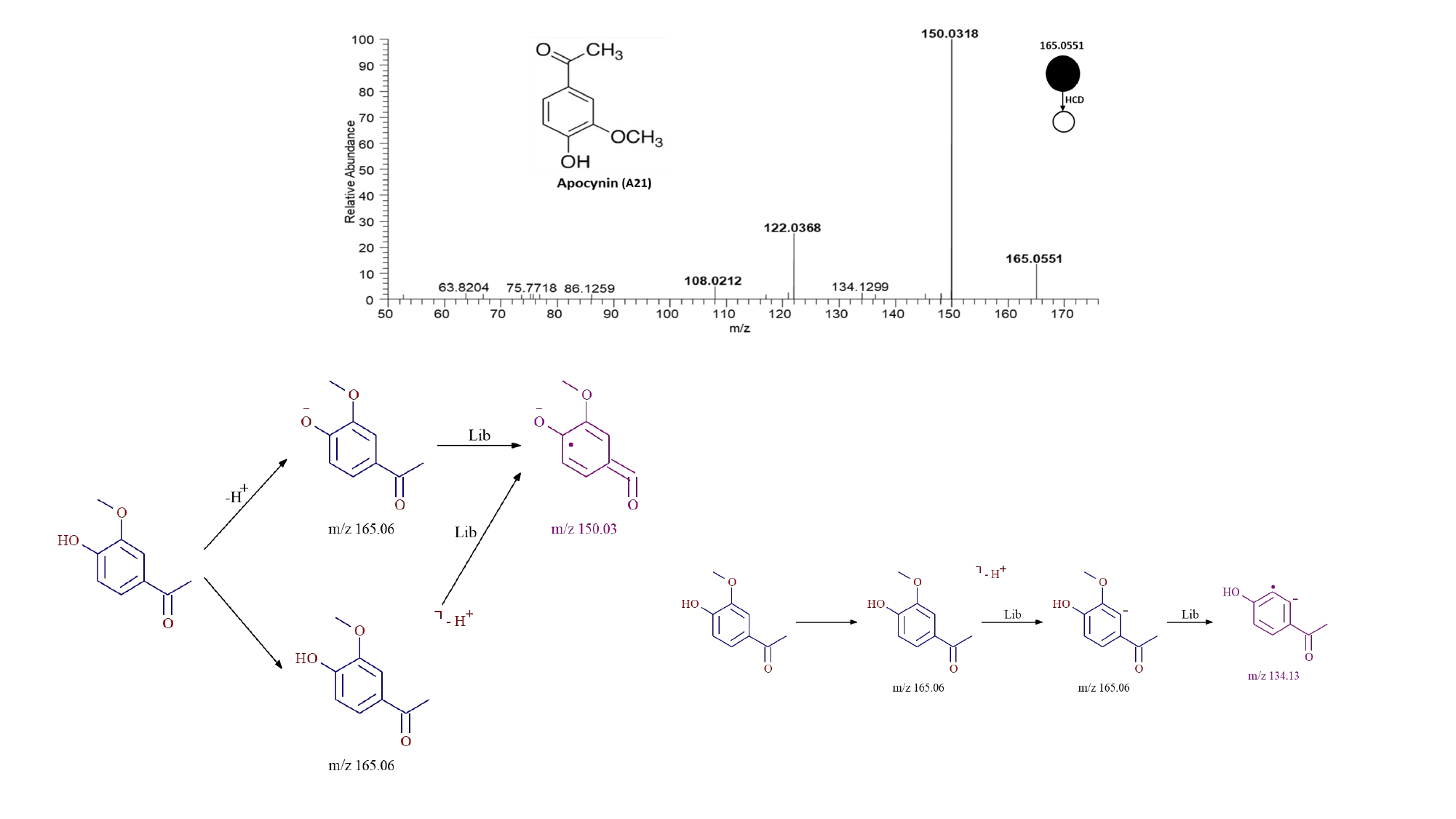

## Slide 33
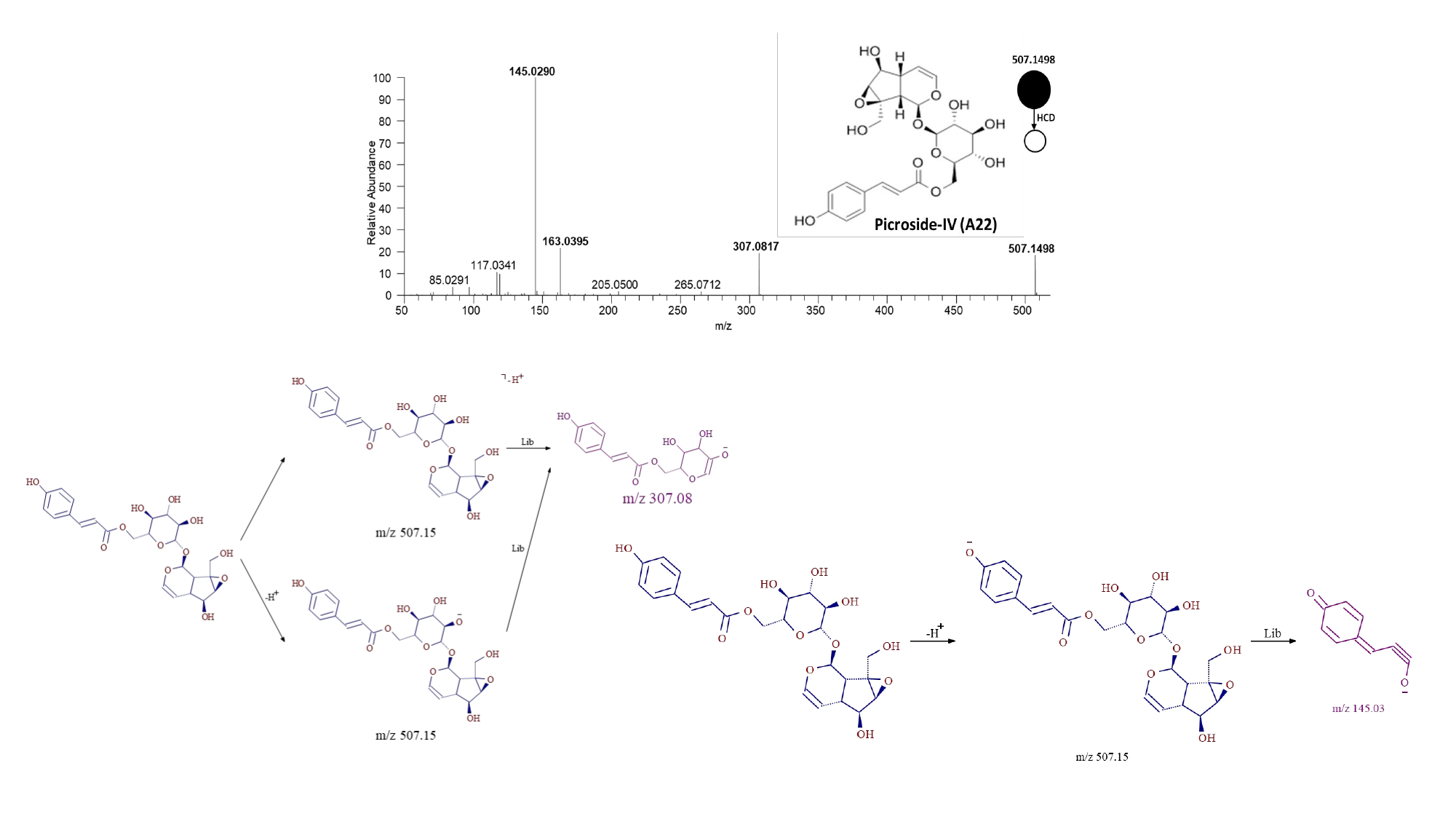

## Slide 34
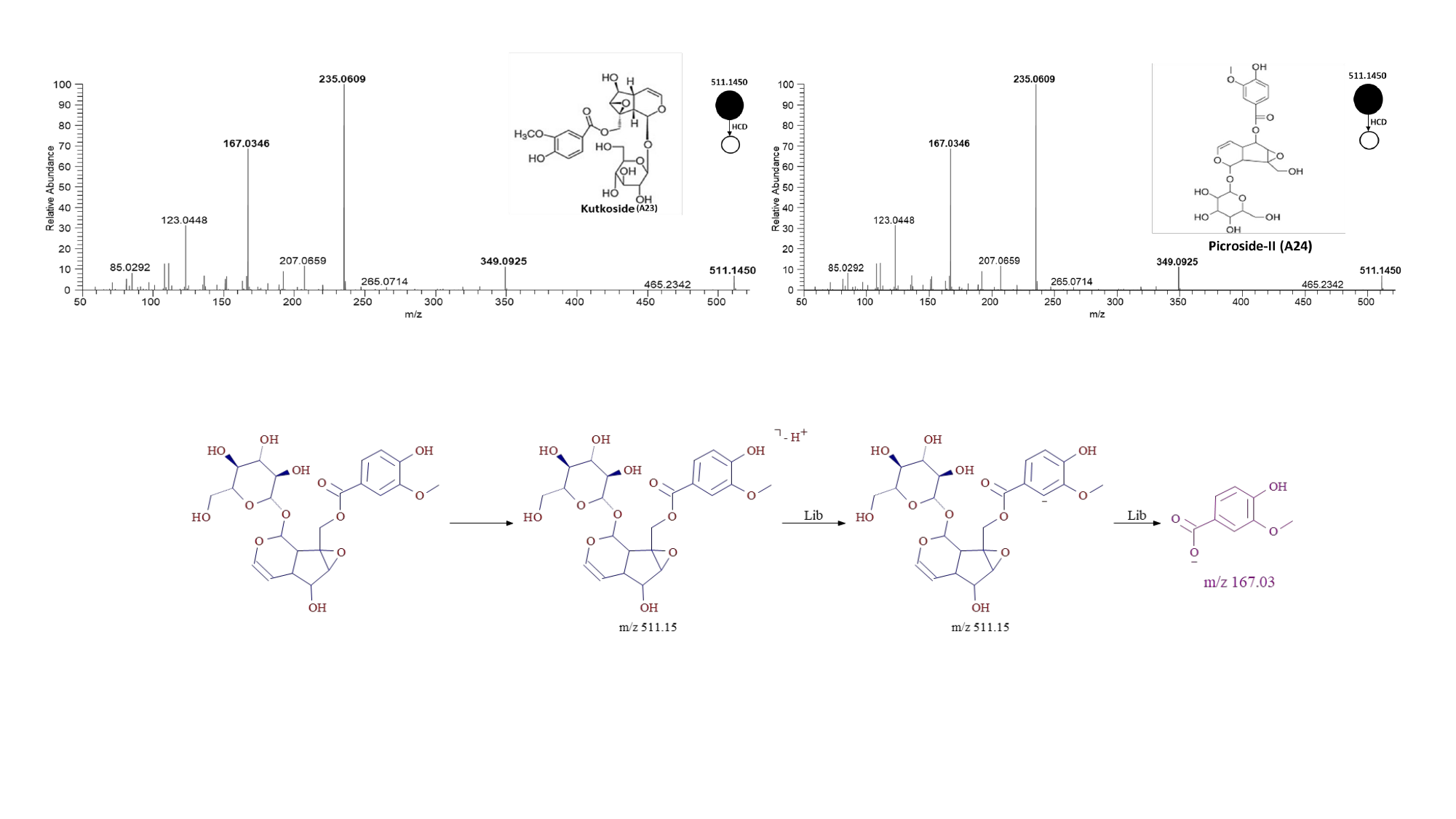

Supplement: Supplementary file 1 [file Presentation1.PPTX]
